# Upcycling Lignin into Porous Hybrid Beads and Sponges for Efficient Removal of Organic and Biological Contaminants from Water

**DOI:** 10.1021/acsmaterialsau.5c00147

**Published:** 2025-12-09

**Authors:** Nicholas Breitkreuz, Tatyana L. Povolotsky, Philip Nickl, Mohsen Adeli, Rainer Haag, Sanjam Chandna

**Affiliations:** † Institute for Chemistry and Biochemistry, Freie Universität Berlin, Berlin 14195, Germany; ‡ Department of Organic Chemistry, Faculty of Chemistry, Lorestan University, Lorestan 68151-44316, Iran

**Keywords:** biopolymer, lignin–chitosan composites, adsorber beads, suspension polymerization, wastewater treatment, antibacterial sponges

## Abstract

Modern industrialization leads to the widespread discharge of pollutants, such as organic dyes and bacterial contamination, into water streams, necessitating efficient and sustainable treatment strategies. To address this challenge, we report the fabrication of biodegradable adsorbents for efficient adsorption of chemical pollutants while reducing the microbial load. Herein, kraft lignin (KL) is cross-linked with chitosan to produce highly porous spherical beads, while EDC/NHS ((*N*-ethyl-*N*′-(3-(dimethylamino)­propyl)­carbodiimide/hydroxysuccinimide) coupling chemistry enabled the formation of microporous sponges and alginate-integrated beads. Cross-linking with chitosan induced a surface charge reversal of lignin from negative (−37 mV) to positive (+51 mV), enhancing its affinity for anionic contaminants. The resulting materials exhibited promising adsorption capacity for an organic pollutant (methyl orange). Furthermore, the composites demonstrated efficient bacterial adsorption, achieving complete adsorption of *E. coli* (∼4 log reduction) using the lignin–chitosan sponge. These results highlight the potential of lignin–chitosan architectures as sustainable and multifunctional materials for advanced water purification.

## Graphical abstract

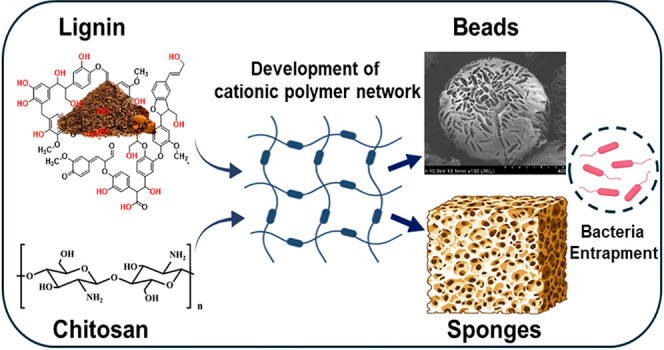

## 1 Introduction

A variety of technologies including electrochemical processes, biological treatment, physical separation, and membrane filtration have been explored for the removal of persistent pollutants (such as dyes, microbial contaminants, perfluoroalkyl substances (PFAS), and heavy metals) from wastewater.^1,2^ However, more affordable and sustainable methods are needed to prevent further ecosystem contamination. In the past decades, development of solid adsorbent materials, which operate through physisorption and chemisorption mechanisms, has attracted significant attention. Although different materials such as activated carbon, zeolites, and metal–organic frameworks (MOFs) have shown high adsorption capacity for different pollutants, they often suffer from limitations including poor biodegradability, high cost, and mechanical instability over extended use.^3^ Eliminating both microbial and chemical pollutants from water bodies is a crucial step toward maintaining safe and clean aquatic environments. Large amounts of organic dyes are known to be carcinogenic and poorly biodegradable, whereas inadequately treated urban wastewater represents a source of microbiological health risk through water leaching, runoff, or aerosolization.^4^

Lignin, a complex polyphenolic biopolymer primarily generated as a byproduct of the pulp and paper industry, is the second most abundant natural polymer after cellulose.^5^ Despite the global production of 50–70 billion tons annually, only around 2% of lignin is used for value-added applications, while the vast majority is incinerated for energy.^6^ The highly branched, heterogeneous structure of lignin renders challenges for characterization and functionalization; however, its rich abundance in polyphenolic functional groups offers significant potential for chemical modification.^7^ Moreover, lignin possesses inherent antioxidant, UV-blocking, and antibacterial properties, further enhancing its value as a sustainable material for environmental applications.^8–11^

The biodegradability and biocompatibility of lignin make it a suitable platform for development of eco-friendly adsorbents. It can be degraded by thermophilic microfungi and actinomycetes into nontoxic byproducts and generally exhibits low cytotoxicity—an important consideration in water treatment systems.^12,13^ Chemical modification strategies including cross-linking,^14^ grafting,^15^ and blending have been employed to incorporate other functional groups such as amines in its structure and enhance its adsorptive performance.^16^ For example, Ge et al. have grafted methylamine onto alkaline lignin (AL) via the Mannich reaction, increasing its nitrogen content to 8.3% and achieving a Pb^2+^ adsorption capacity of 60.5 mg/g.^17^ In another study, Nair et al. have developed AL–chitosan composites that improved the adsorption capacity for Remazol Brilliant Blue R (RBBR) by up to 33% compared to chitosan alone.^18^ Huang et al. have described the synthesis of N-doped microporous carbon using lignosulfonate (chemically different from lignin used in this work) and chitosan. These composites were utilized for *p*-Nitrophenol (PNP) adsorption from water.^19^ In a recent study by Sun et al., phosphorylated chitosan–lignin composites were developed via a Mannich reaction. These composites demonstrated good adsorption capacity and selectivity for PB­(II) and Cu­(II).^20^ A review of the literature revealed that the majority of previous studies concentrated on the adsorption of dyes and heavy metals from wastewater.

Chitosan, a linear polysaccharide derived from chitin, consists of β-(1→4)-linked glucosamine and N-acetyl-glucosamine units.^21^ It can be found in the cell wall of fungi and the exoskeleton of arthropods such as insects, crustaceans, and arachnids.^21^ Its water solubility and biological activity arise from the protonation of amine groups, although solubilization of variants with molecular weights higher than 30 kDa often requires acidification.^22^ The biodegradability, biocompatibility, and cationic nature of chitosan make it widely applicable in food packaging, biomedical devices, and wastewater treatment.^23^ Elzahar and Bassyouni demonstrated that chitosan–polyacrylamide microbeads could remove up to 94% of Direct Blue 78 dye at an initial concentration of 50 mg/mL by optimizing chitosan content.^24^ In a recent work, Marin et al. have cross-linked chitosan with furfural and glutaraldehyde via dynamic imine bonds. These have led to the development of a hydrogel with mean pore diameter between 15 and 35 μm. The authors suggested that such hydrogels can be used as sanitizers (biocidal agents for topical applications).^25^ In another work by Hadizadeh et al., chitosan-based hydrogels cross-linked with gelatin and metal ions exhibited high antibacterial activity against *Staphylococcus aureus* and *Escherichia coli*­(*E. coli*)*.* ^26^

In this work, we present multifunctional materials based on kraft lignin (KL) and chitosan, designed for the removal of both organic and biological contaminants from wastewater. Three cross-linking strategies were developed to create the aforementioned lignin–chitosan materials. Amide coupling was used to create microporous sponges or beads, whereas inverse-suspension polymerization of lignin and chitosan using glutaraldehyde facilitated the formation of porous beads. The efficiency of the synthesized lignin–chitosan materials for the adsorption of wastewater contaminants including negatively charged organic dyes and bacteria was investigated.

## 2 Experimental Section

### 2.1 Chemicals and Materials

Softwood kraft lignin BioPiva 100 was provided by UPM Biochemicals (Finland). Chitosan (600,000–800,000 kDa) was purchased from Thermo Scientific (Germany). 1-Ethyl-3-(3-(dimethylamino)­propyl)­carbodiimide hydrochloride (EDC·HCl) was purchased from abcr GmbH (Karlsruhe, Germany). Sodium alginate (SA) (120–190 kDa from brown algae), glutaraldehyde (50 wt % in H_2_O), 1-ethyl-3-(3-(dimethylamino)­propyl)­carbodiimide (EDC), *N*-hydroxysuccinimide (NHS), *N*-hydroxy-5-norbornene-2,3-dicarboxylic acid imide, chromium­(III) acetylacetonate, chromium­(III) acetylacetonate, 1-methylimidazole (>99% purity), and sorbitan monostearate (Span 60) were purchased from Sigma-Aldrich (Germany). Succinic anhydride (>99% purity), dimethyl sulfoxide (DMSO), and cyclohexane were purchased from Fischer Scientific (Waltham, MA, USA), whereas the anionic dyes methyl orange (MO) and methylene blue (MB) were purchased from Acros Organics (Geel, Belgium) and Merck (Darmstadt, Germany), respectively.

### 2.2 Characterization Methods

#### 2.2.1 Fourier-Transform Infrared (FTIR) Analysis

FTIR spectra were recorded using an Alpha II FTIR spectrometer (Bruker Optik GmbH) with a platinum attenuated total (ATR) unit equipped with a monolithic diamond crystal. Prior to measurements, the samples were ground into powder (where possible) and the adsorption spectra of the samples were measured in the range of 400 cm^–1^ to 4000 cm^–1^ wavenumbers. Baseline correction was performed using OriginPro 2025 software (OriginLab Corporation, Northampton, MA, USA) and spectra corresponding to lignin-containing samples were normalized to the peak corresponding to the aromatic C=C backbone of lignin.

#### 2.2.2 Thermogravimetric Analysis (TGA)

TGA experiments were performed using a TGA 8000 thermogravimetric analyzer (PerkinElmer) under a nitrogen (N_2_) atmosphere. TGA ceramic crucibles (PerkinElmer) were used to contain the samples, whereupon it was heated to 50 °C for 15 min to eliminate surface-adsorbed water, followed by heating until 800 °C at a heating rate of 20 °C/min.

#### 2.2.3 Scanning Electron Microscope (SEM) Analysis

SEM was performed using a ZEISS Sigma 300 VP field-emission Scanning Electron Microscope. The freeze-dried sample was mounted on a coverslip by using a thin carbon and chromium coating. The samples were then coated with colloidal gold particles and allowed to dry at room temperature (25 °C) prior to analysis. The average pore diameter of Chi-Lig-1@40 °C was calculated through the individual measurement of 8 different pores in Figure 2c.

#### 2.2.4 Zeta Potential (ZP) Analysis

ZP measurements were performed using Malvern Zetasizer Nano ZS. Prior to measurement, the samples were prepared by dispersing 1 mg of lignin adsorber materials in 2 mL of 0.1 M acetic acid (pH ≈3 in aqueous solutions). The suspensions were then filtered through a 0.2 μm Regenerated Cellulose syringe filter and sonicated for 15 min prior to measurement. The ZP was determined by taking the average of 3 measurements with an average of 10 scans per measurement.

#### 2.2.5 X-ray Photoelectron Spectroscopy (XPS) Analysis

Near-ambient pressure (NAP)-XPS experiments were performed with an EnviroESCA spectrometer (SPECS Surface Nano Analysis GmbH, Berlin, Germany), equipped with a monochromatic Al Kα X-ray source (Excitation Energy = 1486.71 eV) and a PHOIBOS 150 electron energy. Samples for XPS analysis were prepared on indium foil. The spectra were measured in normal emission, and a source-to-analyzer angle of 55° was used. All spectra were acquired in fixed analyzer transmission (FAT) mode. The binding energy scale of the instrument was calibrated, following a technical procedure provided by SPECS Surface Nano Analysis GmbH (calibration was performed according to ISO 15472). For quantification, the survey spectra were acquired at ultrahigh vacuum conditions (*p* < 1 × 10^–5^ mbar) with a pass energy of 100 eV, and the spectra were quantified utilizing the empirical sensitivity factors that were provided by SPECS Surface Nano Analysis GmbH (the sensitivity factors were corrected with the transmission function of the spectrometer). For charge compensation, the highly resolved XP spectra were acquired under NAP conditions (*p*_H2O_ = 5 mbar) with a pass energy of 50 eV, and the respective data were fitted using UNIFIT 2020 data processing software. For fitting, a Shirley background and a Gaussian–Lorentzian sum function [peak shape model GL (30)] were used. If not denoted otherwise, the L–G mixing component was set to 0.30 for all carbon peaks and 0.40 for all heteroatom peaks. All binding energies were calibrated to the signal observed for the aliphatic C–C bond component (*E*_bind_ = 285 eV) if not stated otherwise.

#### 2.2.6 UV/Vis Spectroscopy Analysis

UV/vis spectroscopy experiments were performed with an Agilent Cary 8454 UV–vis spectrophotometer. The cuvettes used were disposable and were made of polystyrene. The measured wavelengths were within the range of 400–1100 nm. Calibration curves for MO and MB were established by taking the UV/vis absorption spectra at different concentrations. The most intense absorbance for MO within the visible region occurs at 464 nm, whereas it occurs at 665 nm for MB. The absorbance values at these regions were plotted versus dye concentration to obtain a linear curve that follows the Beer–Lambert law, given by the following equation:

1 A=εbC

where *A* is the absorbance, ε the molar extinction coefficient, *b* the path length, and *C* is the concentration of the sample.

#### 2.2.7 ^31^P Nuclear Magnetic Resonance (NMR) Analysis

^31^P NMR measurements were performed on a 500 MHz spectrometer (VNMRS500, Varian Associates, Palo Alto, USA) operating at a proton resonance frequency of 499.9 MHz and a ^31^P resonance frequency of 202.4 MHz, equipped with a 5 mm OneNMR probe. Gravimetric determinations were conducted using an ultramicrobalance (XP2 U/M, Mettler-Toledo, Gießen, Germany).

The sample, internal standard *N*-hydroxy-5-norbornene-2,3-dicarboxylic acid imide (e-HNDI) and relaxation agent Cr­(acac)_3_ were accurately weighed and dissolved in a CDCl_3_/pyridine solvent mixture (1:1.6, v/v). Derivatization was performed following a reported procedure by adding 100 μL of 2-chloro-4,4,5,5-tetramethyl-1,3,2-dioxaphospholane (TMDP) to the solution and shaking for approximately 1 h. ^31^P NMR spectra were acquired by using a 90° pulse angle, a relaxation delay of 10 s, and an accumulation of 256 scans.

#### 2.2.8 Swelling Test

The swelling property of Alg-Chi-Lig-*x* beads in water was tested. Approximately 20 mg of beads were added to 200 mL of Milli-Q water. After 20 h, the beads were removed and gently dabbed with a filter paper to remove surface water, and the weight of the beads was measured. The swelling ratio was calculated as a percentage of the initial weight using the following equation:

2 $$swelling\;ratio\left(\%\right)=\frac{W_{s}{-W}_{d}}{W_{d}}\times 100$$

where *W*_d_ and *W*_s_ are the weight of the dried and swollen beads, respectively.

#### 2.2.9 Determination of the Deacetylation Degree of Chitosan

The deacetylation degree (DD) of chitosan was determined via a titration method adapted from Czechowska-Biskup et al.^27^ Chitosan (0.2 g) was first dissolved in 0.1 M HCl (20 mL), and then 10 mL of H_2_O was added. Under continuous stirring, 0.5 mL of NaOH (0.1 M) was added to the chitosan solution, and the pH was measured. The addition of NaOH was repeated until the pH reached a value of 12.

To calculate the DD, the results were evaluated until the titration reached pH 3 using the following equation:

3 $$f\left(x\right)=\left(\frac{V_{0}+V}{N_{B}}\right)\times \left(\left[H^{+}\right]-\left[{OH}^{+}\right]\right)$$

where *V*_0_ and *V* correspond to the volumes (in mL) of chitosan solution and added NaOH, respectively. *N*_B_ is the concentration of NaOH in mol L^–1^. [H^+^] and [OH^–^] are, respectively, the concentration of hydrogen and hydroxide in mol L^–1^.

The resultant curve was extrapolated to the *x*-intercept, which corresponds to the volume of NaOH at the end point. The DD was then calculated by using the following equations:

4 $$DD\left(\%\right)=\frac{\theta }{\frac{\left(w\times 161\times \theta \right)}{204}+\theta }\times 100$$

5 $$\theta =\frac{\left(N_{A}\times V_{A}-N_{B}\times V_{B}\right)}{1000}$$

where *w* is the weight of the sample in g and *N*_A_ and *N*_B_ are the concentration of the HCl and NaOH in mol L^–1^, respectively. *V*_A_ is the volume of HCl and *V*_B_ is the volume of NaOH in mol L^–1^.

### 2.3 Synthesis

#### 2.3.1 Preparation of Carboxylated Lignin

Lignin (5 g) and succinic anhydride (4.5032 g, 45 mmol) were dissolved in 50 mL of DMSO. To the solution was added 1-methylimidazole (0.2550 g, 3.105 mmol), then stirred (250 rpm) at 60 °C for 24 h. Upon reaction completion, the solution was precipitated in 200 mL of deionized water. The solids were collected through centrifugation (10 min, 10,000 rpm), filtered, and washed with deionized water. Finally, the carboxylated lignin was oven-dried at 40 °C to yield light-brown solids (mass balance = 30%)

#### 2.3.2 Preparation of Chitosan–Lignin Composites (Chi-Lig-*x*) via EDC/NHS Coupling

Chitosan (0.5 g) was dissolved in 0.1 M acetic acid (50 mL) to make a 1 wt % chitosan solution. The solution was stirred for 24 h in room temperature and sonicated briefly to ensure complete dissolution.

To 10 mL of DMSO was added raw lignin (0.5 g), followed by stirring at room temperature until complete dissolution. EDC and NHS were added to the lignin solution. The exact quantities used of EDC and NHS to obtain Chi-Lig-*x* copolymers were as follows: at different Chi/KL ratios (denoted as “*x*”) of 1, 0.8, 0.6, 0.4, and 0.2, the amount of EDC/NHS used was 38.8/57.5 mg, 31/46 mg, 23.3/34.5 mg, 15.5/23 mg, and 7.8/11.5 mg, respectively. The lignin solution was stirred (250 rpm) at room temperature for 30 min. Afterward, the lignin solution containing activated ester leaving groups was added slowly to the chitosan solution. The mixture was stirred (250 rpm) and brought to either 23 or 60 °C for a reaction time of 4 h. Upon reaction completion, the suspension was purified via dialysis against Milli Q water for 48 h, with 4 changes of Milli Q water. Finally, the suspension was freeze-dried to yield brownish sponge-like solids.

#### 2.3.3 Preparation of Chitosan-Carboxylated Lignin Composites (Chi-cLig-*x*) via EDC/NHS Coupling

Chitosan (0.5 g) was dissolved in 0.1 M acetic acid (50 mL) to make a 1 wt % chitosan solution. The solution was stirred for 24 h at room temperature and sonicated briefly to ensure complete dissolution.

Various amounts of EDC·HCl/NHS were added to a solution of carboxylated lignin (0.1 g) dissolved in DMSO (2 mL) to obtain Chi-cLig-*x* (where “*x*” denotes the ratio of EDC·HCl relative to 9.58 mg) copolymers. The exact quantities of EDC·HCl/NHS used were 9.58/11.5 mg, 23.95/28.75 mg, and 28.74/34.5 mg, respectively. The lignin solution was stirred (250 rpm) at room temperature for 30 min. Afterward, the lignin solution containing activated ester leaving groups was added slowly to the chitosan solution (10 mL). The mixture was stirred at 23 °C for a reaction time of 4 h. Upon reaction completion, the suspension was purified via dialysis against deionized water for 48 h, with 4 changes of deionized water. Finally, the suspension was freeze-dried to yield brown sponge-like solids.

#### 2.3.4 Preparation of Chitosan–Lignin Beads (G-Chi-Lig-*x*%) via Inverse-Suspension Polymerization

Chitosan (0.5 g) was dissolved in 0.1 M acetic acid (25 mL) to make a 2 wt % chitosan solution. The solution was stirred for 24 h at room temperature and briefly sonicated to ensure complete dissolution.

Lignin (0.5 mg) in Milli Q water (10 mL) was sonicated for 45 min to get a fine suspension. Afterward, the lignin solution was mixed with the chitosan solution for 20 min (200 rpm). Separately, a setup combining an overhead stirrer with a centrifugal stirrer and a three-necked 250 mL round-bottom flask was used as the reactor for the inverse-suspension polymerization of lignin with chitosan. To the three-necked round-bottom flask was added 77 mL of cyclohexane. Span 60 (0.77 g) was dissolved in cyclohexane at 50 °C.

The lignin/chitosan mixture was added slowly to cyclohexane and stirred (200 rpm) for 15 min until the water and oil phases were well mixed, then glutaraldehyde was added dropwise at 0.5 mL (2.645 mmol) or 1 mL (5.29 mmol) to facilitate cross-linking for 2 h at 50 °C to yield G-Chi-Lig-*x*% composites with either 33% or 66% cross-linking density. Finally, the small brownish beads were filtered under suction and washed 3 times with cyclohexane, followed by 3 times with Milli Q water. The beads were purified with dialysis over 48 h to remove any unreacted glutaraldehyde, then dried using lyophilization.

#### 2.3.5 Preparation of Chitosan–Lignin–Alginate Beads (Alg-Chi-Lig-*x*) via the Ionic Gelation Method

Alginate beads containing lignin and chitosan were prepared. Two separate beads were produced using different compositions of Alg/Chi/Lig.

Sodium alginate (315 or 450 mg) and chitosan (500 or 750 mg) were added to 35 mL of Milli Q water for 24 h at room temperature and sonicated briefly to create a homogeneous dispersion. Separately, lignin was dispersed in 10 mL of Milli Q water. EDC (38.8 mg, 0.25 mmol) and NHS (57.5 mg, 0.5 mmol) were added to the lignin solution, and the mixture was stirred (200 rpm) for 30 min. The lignin solution was added slowly to the slurry containing sodium alginate and chitosan and allowed to react for 4 h at 40 °C while stirring (250 rpm).

The slurry was cooled with tap water, and then using a stopper, drops of lignin, chitosan, and alginate mixture were dropped into a readied solution of 2 wt % CaCl_2_ in 0.1 M acetic acid, where gelation rapidly occurs to form solid beads. The beads were left within the CaCl_2_ for 24 h to ensure complete gelation, and then the beads were filtered and washed 5 times with copious amounts of Milli Q water. The beads were frozen and lyophilized for drying to yield either Alg-Chi-Lig-*x*-1 or Alg-Chi-Lig-*x*-1.25 (the “*x*” in the sample names denotes the relative amounts of chitosan used compared to lignin) beads of approximately 0.7 cm diameter.

### 2.4 Dye Adsorption Experiments

The lignin adsorber materials were tested for their adsorption behavior in the presence of methyl orange (MO) or methylene blue (MB). This was done by preparing dye solutions of different concentrations and changing the conditions of the pH and time (as described in further subsections) Unless otherwise stated, dye adsorption experiments were conducted by incubating 1 mg of sample in 10 mL of MO solution (*C*_0_ = 150 mg/L) at room temperature for a time of 24 h (150 rpm). After completion of the reaction, the equilibrium dye concentration was measured by centrifuging a certain volume of supernatant from the dye–lignin mixtures at 10,000 rpm (10 min). A UV/vis spectrophotometer was used to measure the concentration of the dye solution by comparison to the calibration curves.

The decolorization efficiency was calculated using the following equation:

6 $$decolorization\;efficiency\left(\%\right)=\frac{C_{0}-C}{C_{0}}\times 100$$

where *C*_0_ and *C* are, respectively, the initial and final concentrations of the dye solution before and after the adsorption tests.

#### 2.4.1 Adsorption Kinetics

The kinetics of the adsorption process were evaluated by adding 25 mg of adsorbents to 250 mL of MO solutions (*C*_0_ = 150 mg/L). The reaction proceeded at 150 rpm under room temperature. 250 μL of the suspensions at the top were collected at different time intervals, from 0 to 30 min for the Chi-Lig-*x* samples and 0 to 300 min for the G-Chi-Lig-*x*% and Alg-Chi-Lig-*x* samples. The timeframes were chosen based on the time required for complete adsorption to take place, which were determined through pretests.

#### 2.4.2 Adsorption Isotherm

The initial concentration of MO solutions was changed from 20 to 150 mg/L. 1 mg of adsorbent was added to 10 mL of MO. The reaction proceeded at 150 rpm under room temperature. 250 μL of the suspensions at the top were collected after 2 h for Chi-Lig-*x* and 24 h for the G-Chi-Lig-*x*% and Alg-Chi-Lig-*x* samples.

The adsorption capacity (*q*_e_) in mg/g is calculated as follows:

7 $$q_{e}=\frac{\left(C_{0}-C_{e}\right)}{m}\times V$$

where *C*_0_ and *C*_e_ are both in the units of mg/L, *m* is the mass of the adsorbent in mg, and *V* is the volume of the dye solution in *L*.

#### 2.4.3 Effect of Initial pH

The influence of pH on the adsorption of MB solutions was determined by adjusting the dye concentration to 75 mg/L at different pH (4, 5, 6, 8, and 9) using 0.05 M HCl. 1 mg of adsorbents was added to 10 mL of MO at room temperature and stirred for 24 h (200 rpm).

### 2.5 Antibacterial Tests

*Escherichia coli* AR3110 K-12 derivative was streaked out on lysogeny broth (LB) agar plates and allowed to grow overnight at 37 °C. A single colony of *E. coli* was then inoculated in 5 mL of LB and allowed to grow to the exponential growth phase (OD_600_ 0.6) for approximately 2–3 h at 37 °C in shaking conditions. The bacterial culture was then diluted to 0.01 OD_600_ concentration, and then 500 μL of this diluted culture was added to a 12-well plate. The solid adsorber materials that were UV sterilized were then added at various quantities to form a final concentration of 1.33, 4, 6, 8, and 10 mg/mL. The plates were placed in an incubator at 37 °C under continuous shaking for 24 h.

The CFU assays were conducted as follows: In a 96-well plate filled with 180 μL of PBS buffer, 20 μL of the incubated compound-bacterial solution was added and mixed. The solutions were 1:10 serially diluted 8 times. Afterward, 20 μL of the diluted bacterial solution were dropped onto LB-agar, dried, and incubated at 37 °C. Finally, using a colony counter, the CFU/mL could be calculated by applying the following formula:

8 $$\frac{CFU}{mL}=\frac{number\;of\;colonies\times dilution}{volume\;plated}$$

The remainder of the experimental culture was transferred to 1.5 mL Eppendorf tubes. Samples were spun down at 13,000 rpm in an Eppendorf table centrifuge, the LB supernatant was thrown out, and 400 μL of DPBS was added. 1 μL of SYTO 9 and 1 μL of propidium iodide were added to each Eppendorf tube, mixed by vortexing, and allowed to react for 30 min in dark conditions at room temperature. 20 μL was then placed on microscope slides and imaged using a stereomicroscope (ZEISS), using 100× in oil magnification. Captured images were processed by using Zen software (Zeiss).

## 3 Results and Discussion

The geometry of the adsorber materials may significantly influence the fluid flow characteristics and mechanical properties of the material. In particular, sponge-like structures may facilitate faster adsorption through rapid adsorption kinetics, whereas bead-shaped adsorbers exhibit improved mechanical properties and are more suitable for packing in a fixed-bed column. The motivation behind this research is to establish and compare different lignin–chitosan systems and determine the optimal approach for developing an efficient adsorber material that also exhibits antibacterial activities. The three cross-linking methods are described in Table 1.

**Table 1:** Description and Comparison of the Various Cross-Linking Strategies Employed in This Work

	Method 1 (Sections 2.3.2 and 2.3.3)	Method 2 (Section 2.3.4)	Method 3 (Section 2.3.5)
Morphology	Sponge	Bead	Bead
Cross-linking Strategy	carbodiimide-mediated coupling	inverse-suspension polymerization	ionic gelation
Key Advantages	+simple and fast	+scalable	+scalable
	+direct coupling of chitosan and lignin without a spacer	+control of size and particle size distribution (psd) through adjusting stirring speed and surfactant concentration	+control of particle size through nozzle diameter
	+porous scaffold facilitates mass transfer		+very narrow particle size distribution
			+no toxic chemicals needed

The lignin–chitosan sponge materials were synthesized by reaction between carboxylic groups of the lignin and amine groups of chitosan by using carbodiimide chemistry. To obtain the optimal stoichiometric ratio of lignin/chitosan, the deacetylation degree (DD) of chitosan was first calculated using an acid–base titration method (DD = 88%) (Figure S1 Supporting Information). The reaction first proceeded with the conversion of the carboxylic acid groups of lignin into reactive acylisourea ester intermediates using EDC. These intermediates can rapidly undergo hydrolysis or be converted into a water-soluble urea side product. To improve the efficiency and yield of the reaction, NHS was used in combination with EDC to stabilize the *O*-acylisourea to form the more stable NHS ester. In the final step, chitosan was added to form amide bonds with the newly formed ester to yield the final stable zero-length cross-linked chitosan–lignin composite (Figure 1b).

**Figure 1:**
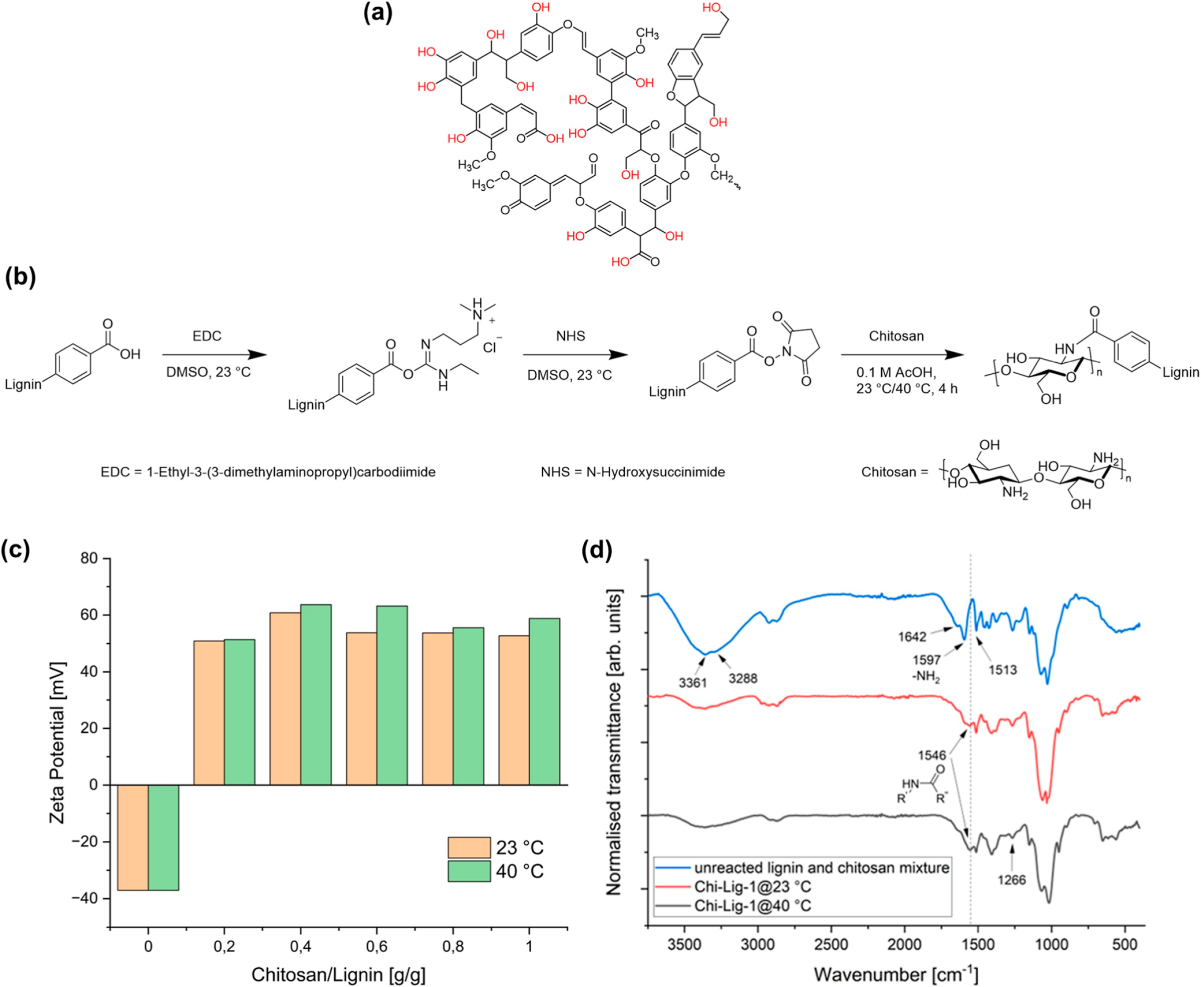
(a) Structure of lignin, adapted from Chandna et al.^33^ (b) Representation of the EDC/NHS coupling reaction between lignin and chitosan. (c) ZP of Chi-Lig-*x* at different reaction temperatures. (d) FTIR spectrum of an unreacted lignin and chitosan mixture, as well as Chi-Lig-*x* sponges.

The reaction conditions were optimized by varying the temperature, amount of cross-linker, and the weight ratio of chitosan/lignin. To ensure the significant conversion of *O*-acylisourea intermediates into the NHS ester, NHS was added at a 1.5-fold excess relative to EDC. As NHS is unreactive to the monomers, the excess did not cause additional side reactions. The quantity of EDC and NHS was calculated based upon the assumption that the raw lignin contains 0.5 mmol of carboxylic acid groups (−COOH) for every gram of lignin. This value was determined through the ^31^P NMR results of KL (Figure S6 and Table S4 Supporting Information). The final product after lyophilization was a sponge-like solid which was insoluble in water and organic solvents. Furthermore, slight adhesiveness to glass and metal surfaces was observed during the handling of the material, suggesting a static charge interaction at the interfaces. This was confirmed by measuring the ZP of the samples (Figure 1c).

The ZP measured for materials synthesized at 23 and 40 °C exhibited similar profiles (Figure 1c). Evidently, there does not seem to be a significant impact of reaction temperature on the surface charges of the materials. At pH 3, the lignin surface exhibited a negative ZP (−37 mV), suggesting that some acidic functional groups (e.g., phenolic –OH and carboxyl (−COOH) were partially deprotonated, contributing to surface negativity. After cross-linking by chitosan (25 wt %), the charge reversal was observed whereupon the ZP value rose above +50 mV. With further increased chitosan content, only a slight increase (approximately 10 mV) in ZP was observed. The DLS measurements of both biopolymers show that lignin particles have a hydrodynamic diameter of 421.5 nm compared to 868.5 nm of the chitosan (Table S1, Supporting Information), thus, even at low quantities of chitosan, it could fully coat the lignin surface. Therefore, high ZP can be achieved at a low chitosan/lignin ratio.

To confirm the success of the cross-linking process, and for comparing the differences in the structure of the final product, FTIR spectroscopy was used to analyze the functional groups present before and after cross-linking. The FTIR spectra of a mixture of unreacted lignin and chitosan as well as Chi-Lig-1 composites formed at 23 and 40 °C are presented in Figure 1d. Upon comparison with the FTIR spectra of raw chitosan (Figure S3 Supporting Information) and KL (Figure S4 Supporting Information), it could be observed that the FTIR spectrum of the chitosan–lignin blend displayed the characteristic bands of both chitosan and lignin, with no appearance of new peaks, indicating that no new chemical bonds were formed during blending. The spectra were normalized using the absorbance band at 1513 cm^–1^, corresponding to aromatic rings of lignin.^28^ Two bands at 3361 cm^–1^ and 3288 cm^–1^ correspond to the NH_2_ groups of the glucosamine units.^29^ The broad absorbance band at 3682 cm^–1^–2989 cm^–1^ is assigned to the hydroxyl groups of lignin. The presence of chitosan was confirmed by the appearing of new bands at 1642 cm^–1^ and 1266 cm^–1^, which is characteristic of amine deformation and C–N stretching of amide III.^30,31^ Interestingly, no signals corresponding to carboxylic acids could be identified through FTIR as a consequence of low carboxylic acid content in KL (Figure S4 Supporting Information). The most indicative sign for the successful conversion of amine to amide during the cross-linking process is represented by the shift of the absorbance band from 1597 cm^–1^ (δ_N–H_ of amine I) to 1546 cm^–1^ (δ_N–H_ of amine II), which suggests a change in the local environment for chitosan’s nitrogen groups (Figure S3 Supporting Information).^32^ The intensity of the absorbance band at 1546 cm^–1^ appears slightly higher in the spectra of Chi-Lig-1 formed at 40 °C compared to room temperature. This means that reaction at 40 °C may yield higher density of cross-linking. Due to more positive surface charge and higher density of cross-linking, Chi-Lig-1@40 °C was further investigated in the next steps.

The thermal stability of Chi-Lig-1@40 °C was evaluated using TGA and differential thermogravimetry (DTG) (Figure S7 Supporting Information). Overall, the mass loss of the Chi-Lig-1@40 °C takes place over three separate regimes. The first weight loss is corresponding to evaporation of water which can be accompanied by dehydration of lignin upon detachment of hydroxyl functional groups (16%).^34^ The evaporation of water was also observed for chitosan and lignin but is negligible compared to Chi-Lig-1. This suggests a higher ability for Chi-Lig-1 to hold greater amounts of water, due to its porous structure. In the second decomposition regime at 273 °C, depolymerization and dehydration of the saccharide rings occurred.^35^ Compared to chitosan, which majorly decomposed at 303 °C, the temperature of decomposition for the chitosan moieties in Chi-Lig-1 was lowered by around 30 °C which can be attributed to reduced hydrogen bonding between the chitosan chains.^36^

The major weight loss occurred during the third regime. The decomposition was centered around 346 °C and proceeded until 800 °C. This is attributed to the pyrolysis of the monomeric segments within lignin, involving the aromatic skeleton as well as the multitude of functional groups such as carbonyl, phenolic, or ether groups. Decomposition steps 2 and 3 overlapped between 247 and 337 °C; therefore, the exact mass loss of either step could not be precisely determined. Interestingly, 40% and 36% of samples remained at 800 °C for lignin and chitosan, respectively, yet for the composite, only 29% of the sample remained. This indicated that the thermal stability of lignin decreases when it is combined with chitosan.

The structure of Chi-Lig-1@40 °C was further characterized by X-ray XPS (Figure S8 Supporting Information). Compared to the XPS results of lignin (summarized in Figure S5 and Table S3 Supporting Information), the C/O ratio of Chi-Lig-1@40 °C decreased from 3.1 to 2.3 due to incorporation of chitosan into the structure of lignin, which contains many –OH, –OR, and –NH_2_ groups. The nitrogen content was undetectable for lignin, but the C/N ratio of the composite was 10.3. In the highly resolved C 1s spectrum of Chi-Lig-1@40 °C (Figure 2a), C–C, C–OR, and C=O bonds were detected at 285 eV, 286 eV, and 288 eV, respectively. In the highly resolved N 1s spectrum (Figure 2b) of the composite, the presence of amine (399.2 eV) and amide/imine (400.2 eV) functional groups was observed, as well as charged quaternary ammonium groups (401.7 eV).

**Figure 2:**
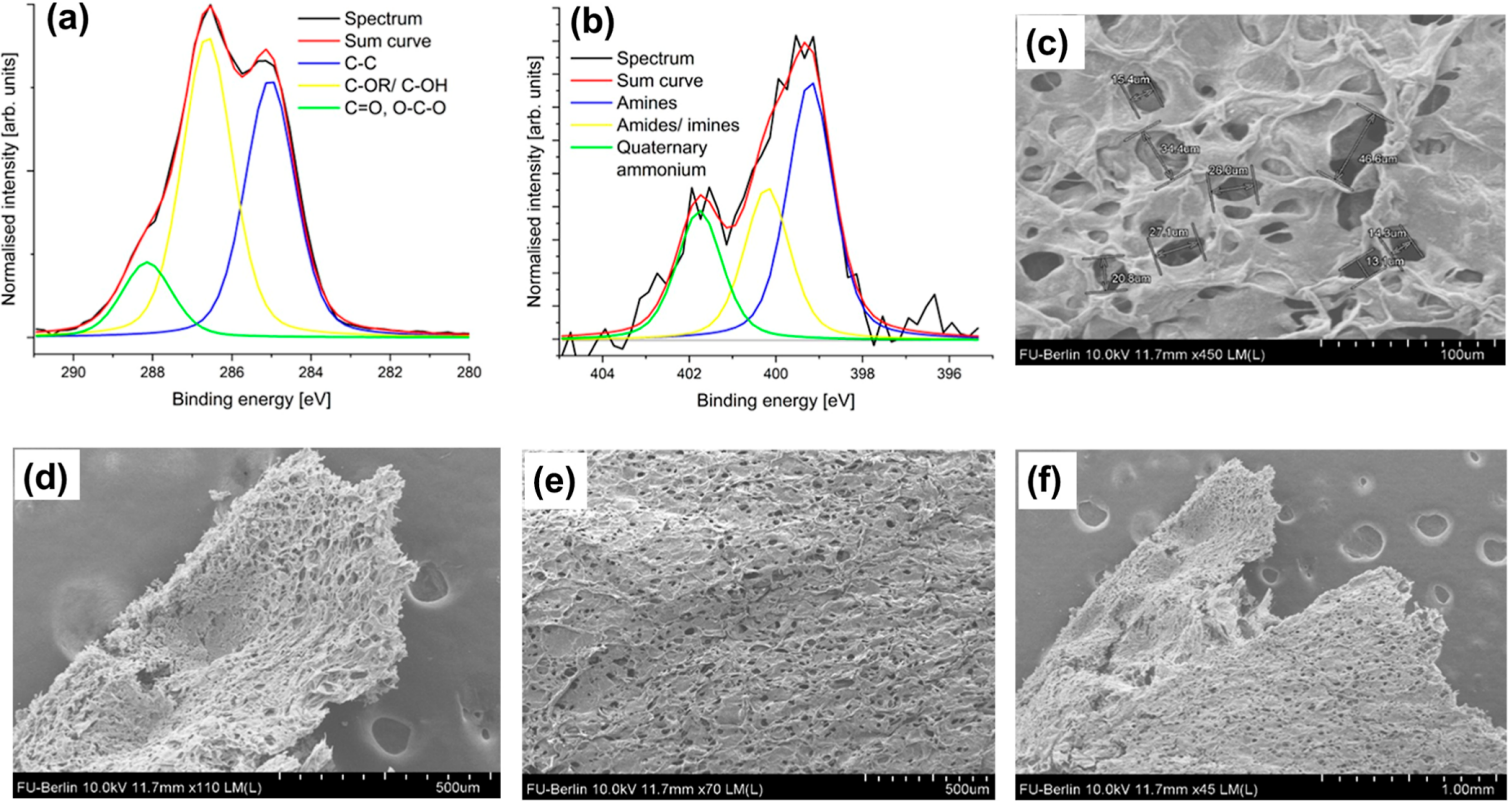
XP spectra of Chi-Lig-1@40 °C. (a) Highly resolved C 1s spectrum and (b) highly resolved N 1s spectrum. (c–f) SEM images of Chi-Lig-1@40 °C at different magnifications (scale bar: (2c) 100 μm, (2d) 500 μm, (2e) 500 μm, and (2f) 1.00 mm).

Since the degree of deacetylation refers to the ratio of amines to amides, the number of amides that were newly formed after the cross-linking could be calculated using eq 9:

9 newlyformedamides(%)=%rel.area(amide)−(100−DD)

Through this method, an increase of 16.26% in amide species among the total nitrogen content of Chi-Lig-1@40 °C was calculated. Additionally, the degree of cross-linking can also be calculated by considering the change in the relative area of the amide signal from the XP spectrum of raw chitosan (Figure S2 and Table S2 Supporting Information) compared to Chi-Lig-1@40 °C. Similar to Chi-Lig-1@40 °C, three peaks were observed in the highly resolved N 1s spectrum of raw chitosan. They correspond to the amine (399 eV), amide (400 eV), and quaternary ammonium species (403 eV) with a relative area of 0.73, 0.23, and 0.04, respectively. In this case, the amide species increased from 23.37% to 28.26%, indicating successful EDC/NHS-mediated cross-linking between lignin and chitosan. Finally, the presence of charged nitrogen is generally observed during the measurements of quaternary ammonium compounds. For chitosan, this refers to the protonated glucosamine units (−NH_3_^+^). SEM showed the sponge-like structure for Chi-Lig-1@40 °C (Figure 2c–f). A high degree of cross-linking could be seen as different parts of the material are interconnected as a complex network. Significant amounts of pores were visible on the surface of the material. The mean pore size of the composite was measured to be 25 ± 4 μm. The existence of the pores may come from either the cross-linking process itself or from the removal of water molecules during the freeze-drying process. For the adsorption of bacteria in later parts of the study, the bacteria can more easily access binding sites due to the increase in surface area resulting from the high porosity. Furthermore, the size of the pores is appropriate for common bacteria like *E. coli* to pass through, as their cell volume is typically within the range of ∼0.4–3 μm^3^.^37^

In another approach to demonstrate the validity of the aforementioned cross-linking strategies, KL was first functionalized with succinic anhydride to considerably increase its carboxylic acid content (Figure S9 Supporting Information). The carboxylated lignin was then reacted with chitosan using various amounts of EDC/NHS. Carboxylated lignin and Chi-cLig composites were analyzed via FTIR (Figure S9 Supporting Information). New peaks corresponding to C=O stretching vibrations of carboxylic acid and ester were observed at 1714 cm^–1^ and 1730 cm^–1^, respectively, for the carboxylated lignin. After cross-linking with chitosan, a decrease in the signal intensity at 1714 cm^–1^ was observed due to the conversion of carboxylic acid functionalities into amide groups. Through these results, we could demonstrate the improved efficiency of the EDC/NHS coupling process of chitosan and lignin. Future work could include prior lignin processing to increase carboxylic acid content for improved cross-linking efficiency.

For the next method, lignin/chitosan composite beads were synthesized via inverse-suspension polymerization using glutaraldehyde as a cross-linker. With this method, additional carbon chains were attached between the lignin and chitosan; thus, unlike EDC/NHS coupling, glutaraldehyde could be described as a “nonzero length cross-linker”. As the reaction involves the simultaneous addition of an amine, non-enolizable aldehyde, and phenol, the Mannich reaction may proceed through the initial formation of the iminium ion, followed up by the electrophilic substitution on the lignin phenol ring at the *ortho*- and *para*-substituted positions, as shown in Figure 3a. Berghuis et al. have previously demonstrated the Mannich reaction of lignin, chitosan, and formaldehyde under basic conditions.^38,39^ Glutaraldehyde was used as a model cross-linker in this work for its well-established reaction mechanism and high efficiency. This enables the evaluation of network structure and functional group retention. Future work will focus on developing greener alternatives compatible with aqueous processing.

**Figure 3:**
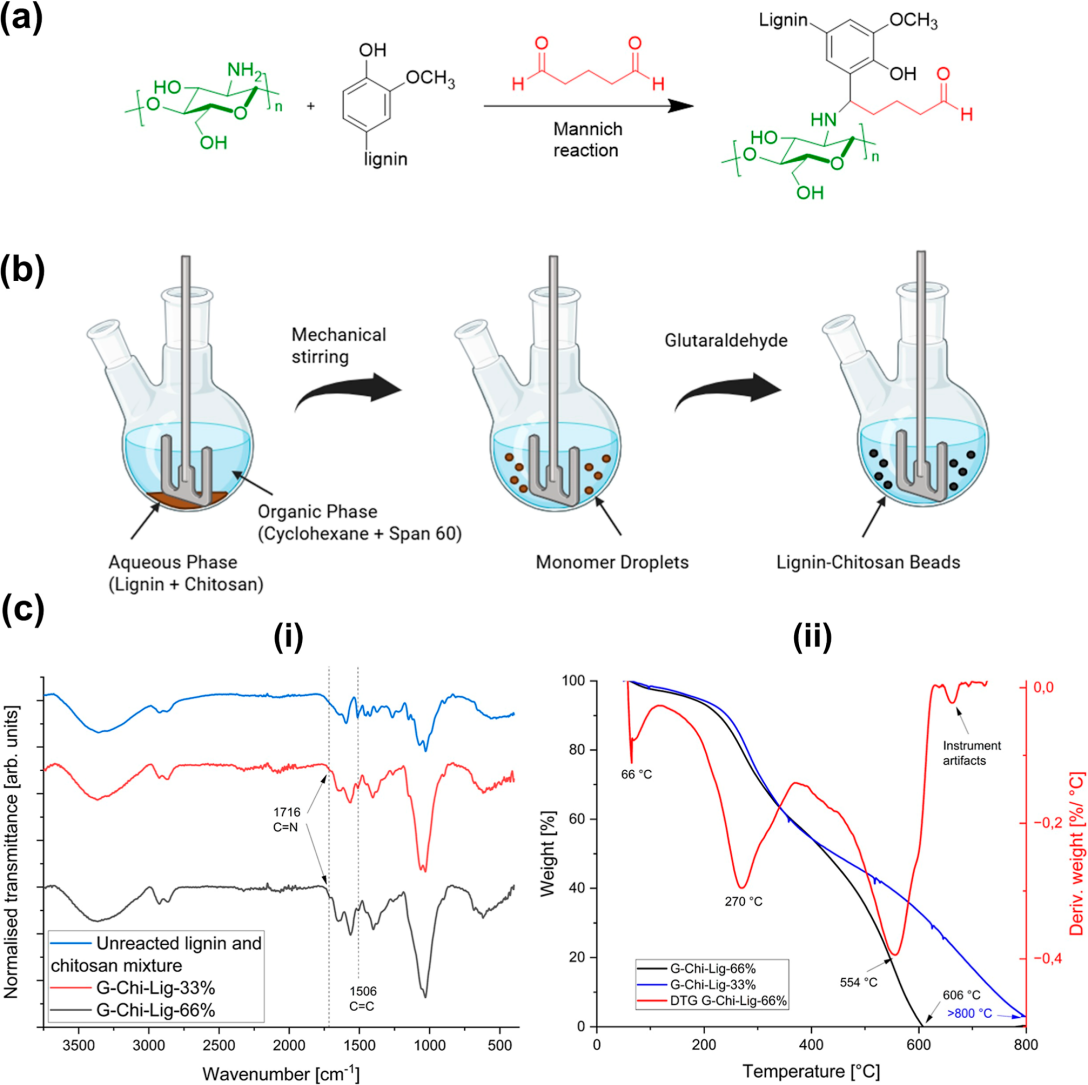
(a) Schematic representation of the synthesis of G-Chi-Lig-*x*% beads using glutaraldehyde as the cross-linker. (b) Inverse-suspension polymerization process for the formation of G-Chi-Lig-*x*% beads “Created in BioRender. Breitkreuz, N. (2025) BioRender.com/0aei05y”. (c) (i) FTIR spectrum of a mixture of unreacted lignin and chitosan, as well as G-Chi-Lig-*x*% beads. (c) (ii) TGA and DTG thermograms of G-Chi-Lig-66% and G-Chi-Lig-33%.

Inverse-suspension polymerization was used to directly form beads during the synthesis process. The method employs an overhead stirrer equipped with a simple paddle. Under mechanical agitation, water (aqueous phase) is dispersed as droplets within cyclohexane (oil phase). The inverse nature refers to the fact that the monomers and cross-linker are soluble within the aqueous phase compared to traditional suspension polymerization. As glutaraldehyde was added to the solution, the monomer droplets were converted into polymer particles as well-defined beads (Figure 3b), which could then be easily isolated via filtration. The advantage of this method is the tunability in the size of beads by changing the agitation speed, solvent ratios, monomer concentration, or cross-linker concentration. As a consequence, each reaction parameter must be well-monitored and -controlled to ensure repeatable results. Initially, a round magnetic stir bar was employed. However, monomer droplets were unstable due to uneven mixing and insufficient sheer force. The reaction vessel was a three-necked 250 mL flask, so that optimal mixing was achieved with a simple overhead stirrer setup. This experimental setup was inspired by the work done by Saidane et al. and Han et al.^40,41^ They have reported the successful preparation of functionalized KL beads by inverse-suspension polymerization.

Span 60 was used to stabilize the inverse suspension and reduce agglomeration by adsorbing at the oil–water interface and providing steric hindrance. Although Span 60 contains hydroxyls that could, in principle, participate in chemical reactions, no evidence of covalent bonding between Span 60 and chitosan was obtained under the mild conditions employed. The observed stabilization is therefore attributed primarily to interfacial adsorption and partial physical entrapment of the surfactant within the polymer matrix.

The water/cyclohexane ratio to achieve optimized beads was 2.22. The effect of cross-linker on the morphology and chemical properties of the beads was investigated by producing two separate products including G-Chi-Lig-66% and G-Chi-Lig-33% that contained 66 and 33 wt % of glutaraldehyde, respectively. The reaction conditions are shown in Table 2.

**Table 2:** Reaction Conditions for the Synthesis of G-Chi-Lig-66% and G-Chi-Lig-33% Beads

	G-Chi-Lig-*x*%	
	*x* = 66	*x* = 33
lignin mass [g]	0.5	0.5
chitosan mass [g]	0.5	0.5
glutaraldehyde [mmol]	5.3	2.65
reaction temperature [°C]	50 °C	50 °C
H_2_O [mL]	35	35
cyclohexane [mL]	77	77
span 60 [mmol]	1.79	1.79
stirring speed [rpm]	200	200

The FTIR spectra of both composites are shown in Figure 3c­(i). The incorporation of lignin within the polymer matrix could be verified by the absorbance band peak at 1506 cm^–1^, which corresponds to the C=C stretching vibrations of the aromatic skeleton of lignin. The successful cross-linking of chitosan using glutaraldehyde was confirmed by the formation of imine bond. The Schiff base could be observed by the existence of the C=N absorbance band at 1716 cm^–1^. Finally, the Mannich reaction could be confirmed by the unchanged intensity for the O–H stretching bands centered around 3000 cm^–1^, as the hydroxyl groups of lignin remained unmodified during the reaction.

The mass losses of G-Chi-Lig-66% and G-Chi-Lig-33% at increasing temperatures were analyzed via TGA (Figure 3c­(ii)). At approximately 700 °C, instrument artifacts caused by external factors generated an additional peak. The first significant mass loss of 2.0% and 2.8% occurred at 66 °C for 33% and 66% cross-linked beads, respectively. This mass loss corresponds to the evaporation of water molecules held within the polymer matrix. The second decomposition step at 270 °C indicates the depolymerization of saccharide rings present in chitosan. Both beads had similar thermal stability until this point, suggesting that modification of the chitosan moieties did not affect the thermal stability of the final polymer. Interestingly, great differences were found for both beads toward higher temperatures at regimes corresponding to the pyrolysis of the monomeric segments within lignin. This occurred at only 554 °C for 66% cross-linked beads, whereas the pyrolysis of lignin took place at 653 °C for the 33% cross-linked beads. Furthermore, complete decomposition of the beads was achieved at 606 °C and above 800 °C for G-Chi-Lig-66% and G-Chi-Lig-33%, respectively. It was hypothesized that increasing the cross-linking of lignin and chitosan should increase its thermal stability, yet the opposite occurred where increased cross-linking significantly decreased its thermal stability.

The XP spectra of G-Chi-Lig-66% are shown in Figure 4a,b and Figure S11, Supporting Information. For raw lignin, no nitrogen could be detected; however, the incorporation of chitosan increased the nitrogen content to 3.35 wt %. The highly resolved N 1s spectrum (Figure 4a) of the composite showed chemical states that are assigned to amine and amide/imine functional groups as well as quaternary ammonium at binding energies of 399, 400, and 402.3 eV. The highly resolved C 1s spectra (Figure 4b) showed C–C bonds (285 eV), C–OR/C–OH bonds (286.5 eV), and C=O bonds (288 eV) matching with the structure of this compound. After cross-linking with chitosan, the C–C:C–O–R/C–O–H ratio decreased from 1.85 to 1.31, due to the incorporation of chitosan, which contains a high content of C–O–R moieties (Figure S11 Supporting Information). Using eq 9, a 20.44% increase of imine groups was calculated, indicating the potential cross-linking of 2 subunits of chitosan. The successful bead formation of G-Chi-Lig-66% was demonstrated by the appearance of a spherical material in the SEM images (Figure 4c­(i–iv)). Some beads were slightly damaged from the filtering and dialysis process; however, the overall structural rigidity was maintained due to covalent cross-linking (Figure 4c­(v)). The average diameter of the beads was 689 ± 50 μm, as measured by taking the average of 6 measurements on the SEM image (Figure 4c­(iv)). The material was found to be highly porous. The average diameter of pores on the surface and center of beads was 39.1 μm ± 5. This is highly advantageous for the adsorption of dyes and bacteria, as the pore sizes were considerably large for the adsorbates to travel through. Furthermore, greater surface area has positive implications on the number of available adsorption sites.

**Figure 4:**
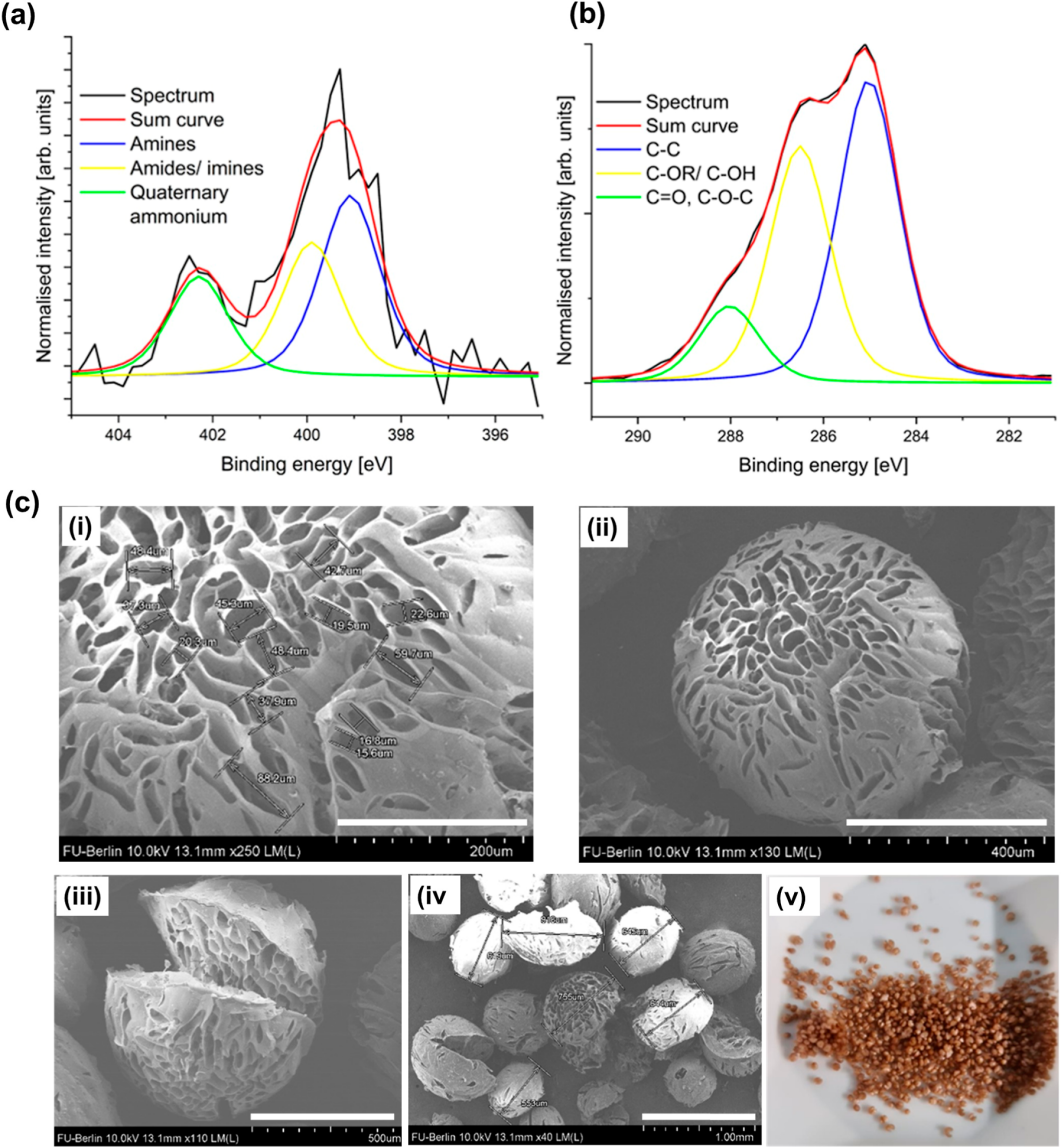
XP spectra of G-Chi-Lig-66%. (a) Highly resolved N 1s spectrum and (b) highly resolved C 1s spectrum. c­(i)–(iv) SEM images of G-Chi-Lig-66% at different magnifications (scale bar: 200 μm, 400 μm, 500 μm, and 1.00 mm). c­(v) Photograph image of the synthesized beads.

Larger beads composed of lignin, chitosan, and SA were formed via the ionic gelation method (Figure 5a). Initially, lignin and chitosan were cross-linked using EDC/NHS coupling chemistry (as previously discussed). Afterward, SA was introduced within the mixture to form a viscous slurry. SA is a biopolymer composed of α-l-guluronic acid and β-D-mannuronic acid, which are linked together in an alternating fashion.^42^ The purpose of the SA during the bead formation process lies in its ability to cross-link with calcium divalent ions through the carboxylate groups present on the alginate backbone. Through electrostatic interactions, the polymer undergoes a conformation change from a sol state to a gel state.^43^ Afterward, the carboxylate–calcium coordination results in a three-dimensional network. The lignin–chitosan polymer could also cross-link with the SA; this involves electrostatic interactions between the carboxylate groups of SA and the ammonium groups of chitosan.^44^ Consequently, due to the dual mechanism of cross-linking, the distinct interchain interactions cause the formation of denser double cross-linked interpenetrated networks.^45^ The cross-linking of alginate with Ca^2+^ ions occurs through the ″egg-box″ model.^45^ Two types of beads with different compositions were made to investigate the effect of biopolymer content on their physicochemical properties (Table 3).

**Figure 5:**
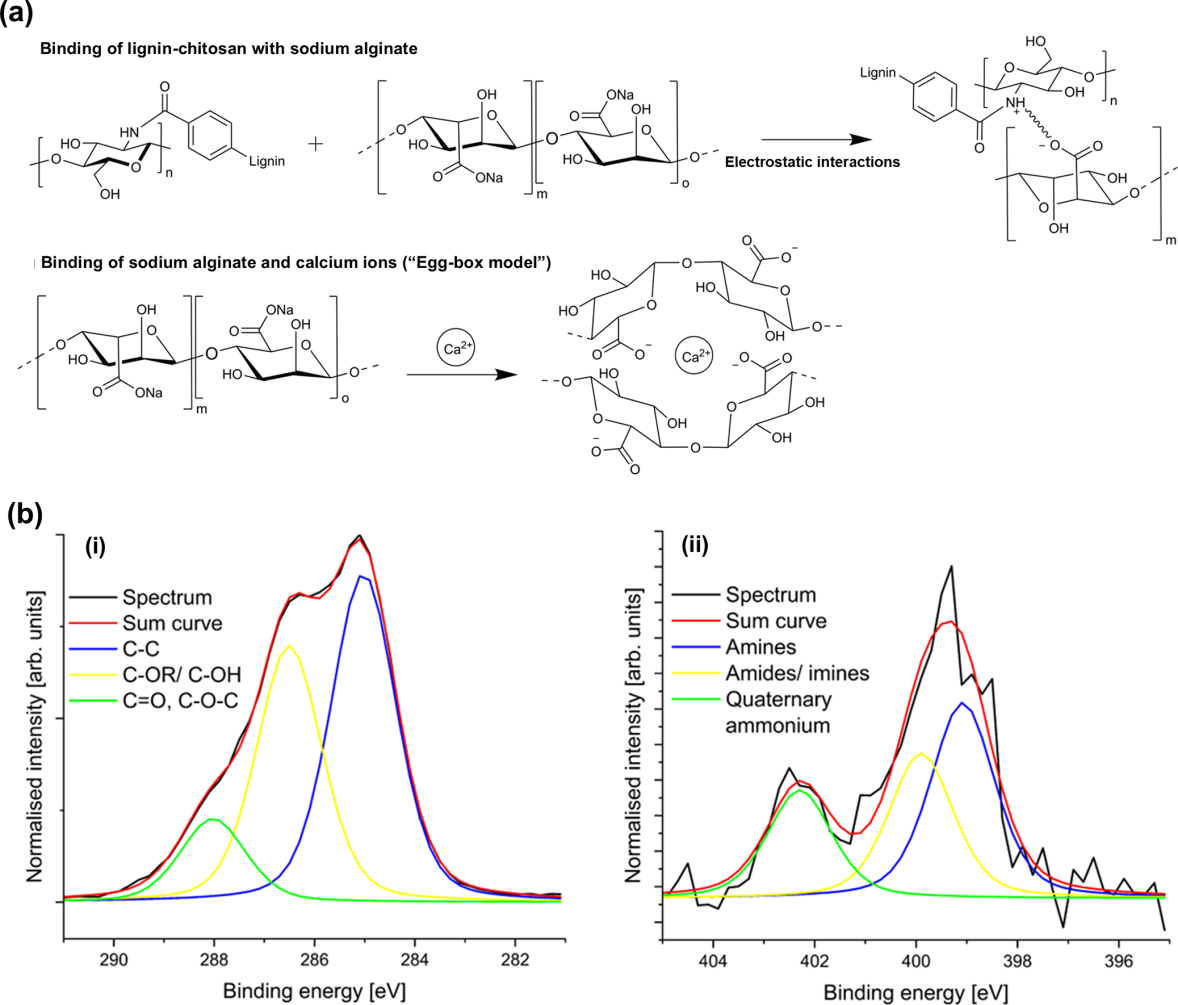
(a) Schematic representation of the synthesis of Alg-Chi-Lig-*x* beads via the ionic gelation method. The chemical structure of sodium alginate was adapted from Foroughi et al.^47^ (b) (i) Highly resolved C 1s spectrum and (ii) highly resolved N 1s spectrum.

**Table 3:** Reaction Conditions, Diameter, Weight, and Swelling Ratio of Alg-Chi-Lig-*x* Beads

	Alg-Chi-Lig-1	Alg-Chi-Lig-1.25
lignin [g]	0.5 (38%)	0.5 (29.4%)
chitosan [g]	0.5 (38%)	0.75 (44.1%)
sodium alginate [g]	0.315 (24%)	0.450 (26.4%)
diameter [cm]	0.7	0.7
weight [mg]	0.8	1.0
swelling ratio [%]	2286 ± 30	3147 ± 2

The beads were named Alg-Chi-Lig-*x* (where “*x*” corresponds to the chitosan/lignin ratio by weight). The elemental composition of Alg-Chi-Lig-1 was evaluated using XPS. In the survey spectrum (Figure S12 Supporting Information), no sodium was found. This indicates that, through either protonation in acidic media or electrostatic interactions with calcium ions, the sodium ions of alginate carboxylate groups have been replaced.^46^ On the other hand, small amounts of chlorine were detected due to contamination during the gelation process. However, calcium ions were also detected, suggesting that the cross-linking of alginate with calcium was successful. The C/O ratio was drastically decreased compared with pristine lignin, changing from 3.1 to 1.9. Even when compared to XPS results of Chi-Lig-1@40 °C (2.25), it could be seen that the incorporation of alginate significantly raised the oxygen content of the Alg-Chi-Lig-1 beads. The incorporation of chitosan into the beads could be observed through the higher amount of N 1s signals in the survey spectrum.

The mass loss of Alg-Chi-Lig-1 and Alg-Chi-Lig-1.25 at increasing temperatures was observed through TGA (Figure S10 Supporting Information). In total, four mass losses were observed. At 84.5 °C, the evaporation of water from the polymer matrix caused a slight mass reduction of 4%. Next, the greatest mass loss was caused by the decomposition of acetylated and deacetylated components of chitosan at 246 °C. This reduced the weight of the sample by 44.9%, which is similar to the relative amount of chitosan added to the reaction mixture (44%). A third decomposition step occurred around the peak of 452.4 °C for the conversion of sodium alginate to Na_2_CO_3_ (mass loss = 24.8%). Again, this value is similar to the relative amounts of sodium alginate added to the reaction mixture (26%). Up until this point, the thermal stability of Alg-Chi-Lig-1 and Alg-Chi-Lig-1.25 was similar as the profile of the curves looked the same; however, for the final decomposition step, the decomposition temperature shifted from 553.2 to 582 °C for the beads with less amounts of chitosan and sodium alginate; furthermore, ash content at the end of measurement was also noticeably more. This mass loss of 22.1% corresponds to the pyrolysis of the monomeric segments within lignin. The greater thermal stability of Alg-Chi-Lig-1 compared to Alg-Chi-Lig-1.25 was due to the incorporation of greater amounts of lignin (38% compared to 29%).

In the highly resolved C 1s spectrum (Figure 5b­(i)), C–C, C-OR/C–OH, and C=O bonds can be detected at 285, 286.5, and 288 eV. Compared to lignin, the C–C and C–O–R/C–O–H ratio decreased from 1.9 to 0.7 due to incorporation of chitosan and alginate, which both possess a chemical composition with high content of C–O–R moieties. More interestingly, in the highly resolved N 1s spectrum (Figure 5b­(ii)) of the composite, nitrogen atoms of amine (399.6 eV) and amide/imine (401 eV) groups as well as charged nitrogen (402 eV) can be seen. The increase in the intensity of the peak component at 401 eV, corresponding to the generation of new amide bonds, was assigned to the successful cross-linking of lignin with chitosan. Overall, a 23.89% increase in amide groups was observed. The morphology of Alg-Chi-Lig-1 was observed through SEM measurements (Figure S13 Supporting Information). The pristine beads display a relatively smooth surface.

Multiple synthetic processes to form lignin–chitosan composites were presented to compare how the structural and thermal properties of these composites can be modified through various cross-linking strategies. XPS results showed that the C–C:C–OH/C-OR ratio of G-Chi-Lig-66% was of a greater value compared to that of the Chi-Lig-1@40 °C composite (1.310 vs 0.858), suggesting that the glutaraldehyde cross-linking process significantly decreased the amount of C–OH and C–OR groups within G-Chi-Lig-66% beads, as well as increased the C–C content. The increase in the C–C content could be well-explained by the addition of carbon chains from the introduced glutaraldehyde backbone; however, the change in C–OH and C–OR groups suggests that glutaraldehyde modification had a great effect on the phenolic units in the lignin.

The ash contents of the composites could be compared by seeing how much of the sample was left at 800 °C during TGA measurements. For Chi-Lig-1@40 °C, 29% of the total weight was left at 800 °C, whereas G-Chi-Lig-66% beads decomposed fully at 606 °C. The lesser cross-linked G-Chi-Lig-33% beads were shown to decompose only when the temperature was raised beyond 800 °C. Finally, ash content was measured as 6.2% and 1.8% for the Alg-Chi-Lig-1 and Alg-Chi-Lig-1.25 beads, respectively. Following these results, it could be concluded that the thermal stability of the materials was as follows (from highest to lowest): (1) Chi-Lig-1@40 °C, (2) Alg-Chi-Lig-1, (3) Alg-Chi-Lig-1.25, (4) G-Chi-Lig-33%, and (5) G-Chi-Lig-66%.

For G-Chi-Lig-66% and Chi-Lig@40 °C composites, the formation of many pores on the material surface was hypothesized to stem from the freeze-drying process, causing sublimation of ice crystals, which could leave behind pores, much like the formation of cryogels. However, despite similar drying processes, no macropores were formed in the Alg-Chi-Lig-1 beads. This may be a consequence of utilizing too concentrated CaCl_2_ during the cross-linking process, as calcium ions have been shown to be able to bind alginate chains together through electrostatic interactions at high concentrations to prevent pore formation.

Overall, all three methods were successful at cross-linking chitosan with lignin. However, each method has its own limitations for scale-up. For carbodiimide-mediated cross-linking, the higher production costs and poor atom economy of the method are a significant bottleneck. Regarding the inverse-suspension process, even though it is a highly scalable process, large amounts of organic solvents are required. Additionally, the process is complex to optimize. Finally, ionic gelation of chitosan–lignin composites through blending with sodium alginate is a particularly effective method to produce beads with the desired size by simply changing the diameter of the nozzle. However, sodium alginate may have negative consequences on the adsorption properties of the beads due to its negatively charged carboxylic acid groups.

After characterization of the lignin–chitosan materials, their ability for dye removal from aqueous solutions was investigated and compared to determine how the difference in morphology (sponge vs bead) of the materials can influence the adsorption processes. Methyl orange (MO) contains one sulfonate group, which imparts it with anionic charge and increased water solubility. The proposed mechanisms for the adsorption of MO onto the lignin–chitosan composite are as follows: significant hydrogen bonding between nitrogen and oxygen atoms of dye, lignin, and chitosan, π–π interactions between the phenyl ring of the dye and aromatic backbone of lignin, and electrostatic interactions between the oppositely charged moieties of the modified lignin and the sulfonate groups of MO.

The adsorption capacity of Chi-Lig-*x* composites for MO was investigated at an initial MO concentration of 150 mg/L (Figure 6a). The adsorption took place over 24 h, and the equilibrium dye concentration in the supernatant was measured using UV/vis spectroscopy. The adsorption capacity of raw lignin was expected to be low due to its negative ZP value. Indeed, the adsorption capacity of lignin was found to be 46 mg/g. However, the adsorption capacity increased with increasing chitosan content in the Chi-Lig-*x* composites. As previously discussed, from the FTIR spectra of Chi-Lig-1@23 °C and Chi-Lig-1@40 °C, it was determined that greater quantities of amides may have formed at 40 °C in comparison to their counterparts synthesized at 23 °C. To observe how this affects the adsorption capacity of the composites, their adsorption capacity was also plotted on the same graph. From the results, it could be seen that reaction temperatures have minimal effects on the adsorption capacity.

**Figure 6:**
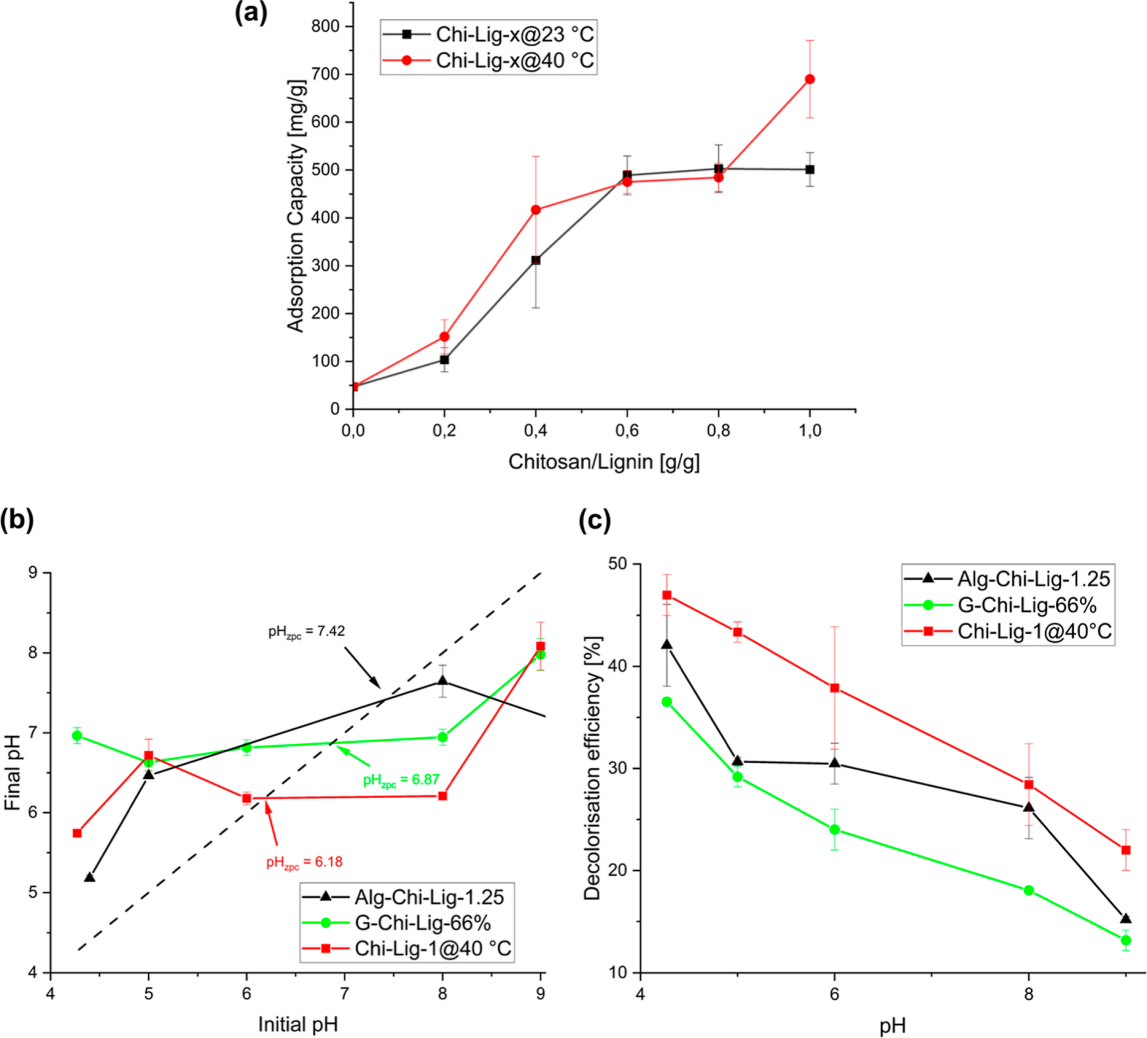
(a) Adsorption capacity of Chi-Lig-*x*@23 °C and Chi-Lig-*x*@40 °C composites at different ″*x*″ amounts. The batch adsorption experiment conditions were 150 mg/L MO concentration, 24 h, room temperature, 0.1 mg/mL dosage. (b) Point of zero charge pH_zpc_ of lignin–chitosan composites in MO, determined by the pH drift method. (c) Decolorization efficiency of lignin–chitosan composites for the removal of MO at different initial pH.

The adsorption equilibrium of Chi-Lig-1@40 °C, G-Chi-Lig-66%, and Alg-Chi-Lig-1.25 for MO was evaluated by using the Langmuir and Freundlich isotherms. These compounds were chosen due to their complete characterization. Furthermore, Alg-Chi-Lig-1.25 is of interest due to the increased chitosan content. The independent variable for these tests was the initial dye concentration, which varied from 20 to 150 mg/L. The composites were shaken within the dye solution to adsorb dye until full saturation was achieved, and an equilibrium concentration could be maintained. For the Langmuir isotherm, *C*_e_/*q*_e_ vs *C*_e_ was plotted, whereas log­(*q*_e_) and log­(*C*_e_) were plotted for the Freundlich isotherm (Figure S14 Supporting Information). Linear regression was done using both data sets, and the determination coefficient *R* ^2^ from each graph then determines whether the adsorption equilibrium follows Langmuir- or Freundlich-type adsorption.

According to Miretzky et al., the linearized Langmuir equation can be expressed as^48^

10 $$\frac{C_{e}}{q_{e}}=\frac{1}{K_{L}}+\frac{a_{L}}{K_{L}}C_{e}$$

where *K*_L_ (mL g^–1^) and *a*_L_ (mL mmol^–1^) are constants for the Langmuir equation. Importantly, the theoretical monolayer saturation capacity, i.e., the maximum adsorption capacity *q*_max_ is determined through the ratio of *K*_L_/*a*_L_. On the other hand, the separation factor *R*_L_, which describes the favorability of the adsorption process, may be represented by eq 11:^49^

11 $$R_{L}=\frac{1}{\left(1+a_{L}C_{0}\right)}$$

*a*_L_ is the Langmuir constant and *C*_0_ is the initial adsorbate concentration. The value of *R*_L_ indicates the nature of the adsorption process: where *R*_L_ = 1: adsorption is linear; 0 < *R*_L_ < 1: adsorption is favorable; and *R*_L_ → 0: adsorption is highly favorable, approaching irreversible adsorption in the limit.

The equation to express the Freundlich equation model is the following:^50^

$$q_{e}=K_{F}C_{e}^{1/n}$$

where *q*_e_ is the amount of adsorbate adsorbed per unit mass of adsorbent (mg/g), *C*_e_ is the equilibrium concentration of the adsorbate in solution (mg/L) after adsorption has reached the steady state, *K*_F_ is Freundlich’s constant ((L mg^–1^)^1/*n*^), and *n* is dimensionless which determines the intensity of the adsorption. Equation 5 could be integrated to its linear form:

12 $$\ln\left(q_{e}\right)=\ln\left(K_{F}\right)+\frac{1}{n}\ln\left(C_{e}\right)$$

The values of *R* ^2^ obtained for Chi-Lig-1@40 °C and G-Chi-Lig-66% were 0.99566 and 0.97558, respectively, for the Langmuir isotherm, whereas the *R* ^2^ values obtained for the Freundlich isotherm were relatively lower. As a result of the good fit to the Langmuir equation, it could be concluded that the adsorption of MO to Chi-Lig-1@40 °C and G-Chi-Lig-66% occurred over a fixed number of sites, where each site only takes up one molecule at a time.^51^ Furthermore, assumptions could be made that all of the sites were energetically equivalent and the interactions between the adsorbed molecules could be neglected. For Alg-Chi-Lig-1.25, the classical Langmuir and Freundlich isotherms were not sufficient to correlate the experimental results with its theory as the obtained *R* ^2^ was poor (0.87987 and 0.90713, respectively). This suggests that there may be mixed adsorption mechanisms that combine physisorption and chemisorption. It may therefore be necessary to consider alternative models as an approach to better describe adsorbate–surface interactions. For example, the Redlich–Peterson model, through combining the features of Langmuir and Freundlich models, may better describe the adsorption process.^52^

The most important parameter that could be elucidated from the Langmuir isotherm is the maximum adsorption capacity *q*_max_. It is calculated using eq 13:

13 $$q_{\max}=\frac{K_{L}}{a_{L}}$$

The experimentally calculated *q*_max_ was 435.76 mg/g for Chi-Lig-1@40 °C, 442.95 mg/g for G-Chi-Lig-66%, and 514.33 mg/g for Alg-Chi-Lig-1.25 (Table 4); however, as the data set of Alg-Chi-Lig-1.25 did not fit well with the Langmuir isotherm, the *q*_max_ would not be accurate. Figure S15 Supporting Information shows the *R*_L_ values at different initial dye concentrations. It could be observed that within 20 mg/L to 150 mg/L, the adsorption process is favorable for all composites; however, adsorption processes involving Chi-Lig-1@40 °C were the most favorable, whereas adsorption processes involving G-Chi-Lig-66% were the least favorable.

**Table 4:** Experimentally Determined Langmuir and Freundlich Parameters

isotherm	parameter	Chi-Lig-1@40 °C	G-Chi-Lig-66%	Alg-Chi-Lig-1.25
Langmuir	*q*_max_ [mg g^–1^]	435.76	442.95	514.33
	*K*_L_ [mL g^–1^]	22.660	27.020	13.887
	*a*_L_ [mL mmol^–1^]	0.052	0.061	0.027
	*R* ^2^	0.99566	0.97558	0.87987
	*R*_L_	0.114–0.492	0.098–0.450	0.197–0.648
Freundlich	*K*_F_ [(L mg^–1^)^1/*n*^]	5.848	5.385	4.782
	*N*	2.358	2.257	1.861
	*R* ^2^	0.98295	0.81551	0.90713

The adsorption mechanism of the tested materials was found to follow the pseudo-second-order (PSO) model (Figure S16 Supporting Information). The *R* ^2^ values from the linear regression of each material were significantly closer to 1 following the PSO model, whereas none of the pseudo-first-order (PFO) models fitted well above 0.8. The results indicate that the rate-determining step of the adsorption of MO involves chemisorption and is dependent on the amount of solute on the surface of the adsorbent.

The PSO rate constant determined from the y-intercept for Chi-Lig-1@40 °C was approximately 6 × 10^–4^ min^–1^ (Table S5 Supporting Information). Compared to G-Chi-Lig-66% and Alg-Chi-Lig-1.25, the determined *k*_2_ values were 2- and 10-fold greater, respectively. The greater rate constant suggests that the adsorption of MO proceeded faster for Chi-Lig-1@40 °C. To explain this, the morphology of the composites must be compared. Since the number of active sites available determines the rate of adsorption, greater porosity as well as surface area of the composites naturally will play a huge role. From previous SEM analysis, it could be seen that the porosity and surface area of the composites follow the order of Chi-Lig-1@ 40 °C > G-Chi-Lig-66% > Alg-Chi-Lig-1.25.

To operate in effluents containing dye, it is necessary for the adsorber materials to retain a good adsorption capacity under different pH conditions. The point of zero charge (pzc) describes the pH at which the net charge on the surface of the material is zero.^53^ To measure the pzc, the lignin–chitosan composites were allowed to adsorb dye until they reached full saturation. The pH of the dye aqueous solution prior to adsorption would then be compared to the pH of the solution after adsorption (Figure 6b). The pzc is the pH at which pH_initial_ = pH_final_. The pzc, measured in the order of the lowest to highest, was as follows: Chi-Lig-1@40 °C (pzc = 6.18), G-Chi-Lig-66% (pzc = 6.67), and Alg-Chi-Lig-1.25 (pzc = 7.42). As expected, these values were near the range of 6.39–6.51, which corresponds to the p*K*_a_ of chitosan.^54^ It could therefore be concluded that the surface charge of the adsorber materials was mainly dependent on the protonation of chitosan.

Next, the removal of MO from aqueous solutions was tested at a pH range of 4–9 for the three different composites (Figure 6c). Remarkably, the decolorization efficiency for G-Chi-Lig-66% and Chi-Lig-1@40 °C decreased linearly with an increase in pH. This is a consequence of the loss of charge upon deprotonation of amino moieties. The highest decolorization achieved by Alg-Chi-Lig-1.25 was at pH 4.27 (42.1%) because of the protonation of hydroxyl groups present in lignin in addition to amine groups of chitosan. The hydroxyl groups would then be able to electrostatically bind to the anionic dye. The decolorization efficiency then dropped significantly at pH 5 (30.7%), as the solution was not acidic enough to significantly protonate the lignin.

Until pH 8, the decolorization efficiency was maintained at a similar level despite the deprotonation of chitosan at higher pH, even when the pH is higher than the pzc. This phenomenon could be attributed to the shrinkage of SA under acidic conditions.^55^ The shrinkage decreases the size of the beads; therefore, the amount of available adsorption sites reduced and the dye particles could not access them. At pH 9, dissolution of the SA and deprotonation of chitosan cause significant reduction in decolorization efficiency (15.2%). To determine if the electrostatic interaction was the principal interaction that governed the adsorption behavior of the composites, the adsorption of MB was also tested. The results are summarized in Figure S17 Supporting Information. For Chi-lig-1@40 °C, the highest decolorization achieved was 47% at pH 4.27.

Finally, the antibacterial activity of the synthesized adsorber materials was tested by incubating the samples in an *E. coli* solution (approximately 10^6^ CFU/mL) for 24 h. *E. coli* was inoculated to simulate bacterial contamination that the material would encounter as part of a wastewater treatment system. For a qualitative estimation of bacterial inhibition, live–dead cell imaging of *E. coli* cells (incubated with different concentrations of Chi-Lig-1@40 °C) was performed (Figure 7a). The SYTO 9 and propidium iodide (PI) stains were used to assess viability of the bacterial cells. SYTO 9 stain is able to permeate bacterial cell membranes and stain the nuclear and chromosome material, and it fluoresces green. PI on the other hand is a DNA intercalating dye that is unable to permeate intact cell membranes, but only damaged or ruptured membranes, therefore it is used to indicate dead or damaged cells and fluoresces red. It was observed that *E. coli* cells fluoresced green in the absence of Chi-Lig-1@40 °C (control). At 2 mg/mL Chi-Lig-1@40 °C, some cells fluoresced red. As the concentration was increased to 4 mg/mL, no cells were observed; this is because the cells were killed initially in the presence of Chi-Lig-1@40 °C. The number of viable bacteria in the supernatant was evaluated by counting the colony forming units (CFU). The results (Figure 7b) showed that G-Chi-Lig-66% and Alg-Chi-Lig-1.25 were initially ineffective as antibacterial agents at 5 mg/mL; however, complete reduction in the bacterial counts (CFU) was observed using Chi-Lig-1@40 °C at 5 mg/mL.

**Figure 7:**
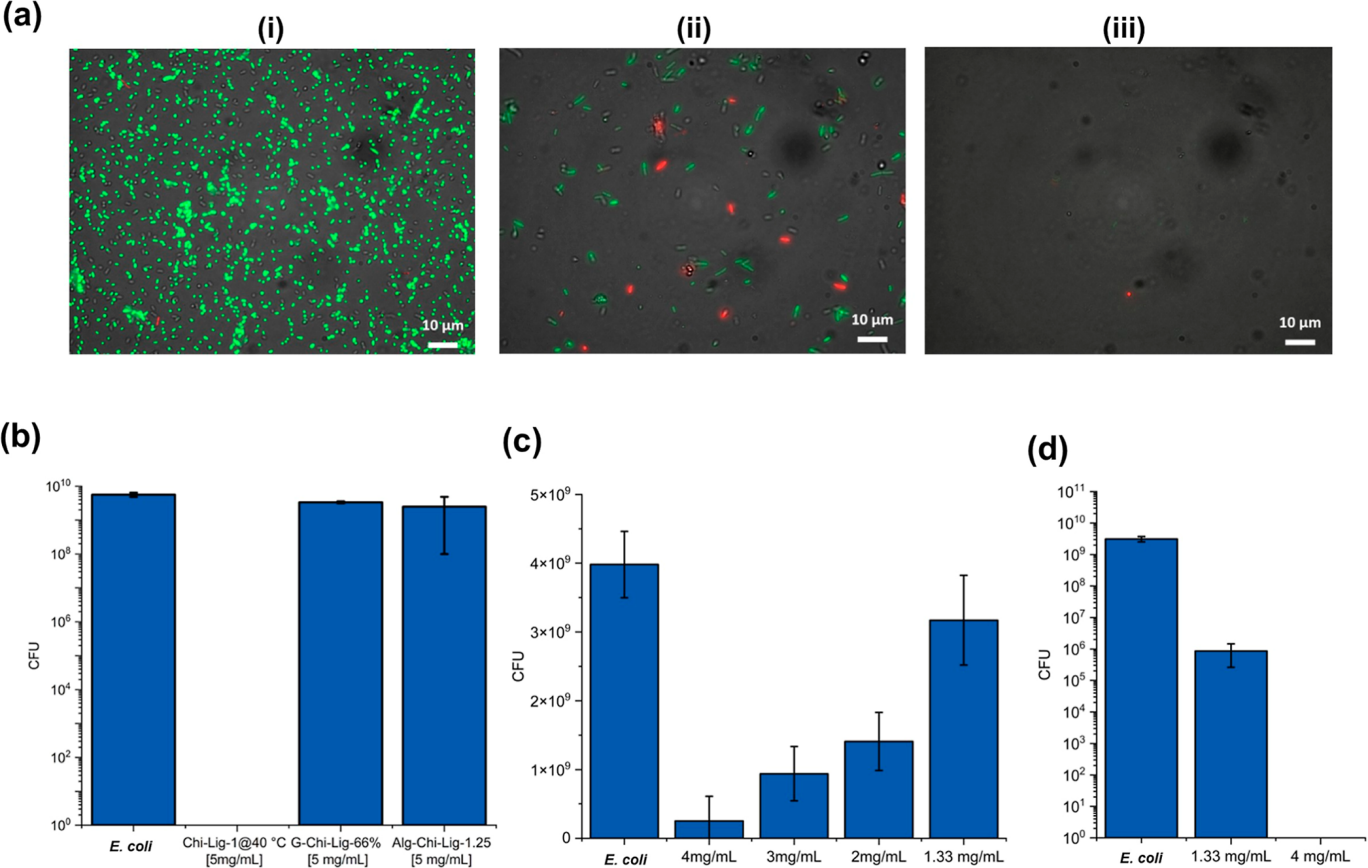
(a) Live–dead cell imaging of (i) control (only *E. coli* cells), (ii) incubation of *E. coli* with 2 mg/mL of Chi-Lig-1@40 °C, and (iii) incubation of *E. coli* with 4 mg/mL of Chi-Lig-1@40 °C. (b) CFU test of *E. coli* contaminated water after treatment with different lignin–chitosan composites. (c) CFU test of *E. coli* contaminated water after treatment with Chi-Lig-1@40 °C at various dosages. (d) CFU test of *E. coli* contaminated water after treatment with seized Chi-Lig-1@40 °C at various dosages.

It is known that the antimicrobial effects of chitosan come from its ability to disrupt the bacterial cell membrane/cell wall.^56^ For Gram-negative bacteria like *E. coli*, the negatively charged lipopolysaccharides (LPS) could be neutralized by the positively charged chitosan; this leads to disruption of the outer membrane (OM), and cell death could be facilitated by the penetration of chitosan into the cell membrane. By increasing the porosity and surface area of the adsorber materials, the contact between chitosan and LPS could be enhanced. Therefore, Chi-Lig-1@40 °C with the highest surface area among synthesized composites showed a better bacterial adsorption and antibacterial activity at a concentration of 5 mg/mL. To gain further information regarding antibacterial activity of lignin–chitosan composites, a more detailed analysis of the adsorber–bacteria interactions is required.

Further, the dose-dependent inhibition study of Chi-Lig-1@40 °C was conducted against *E. coli*. The dosage of Chi-Lig-1@40 °C was varied from 0 to 4 mg/mL to determine the minimum inhibitory concentration (MIC). For Chi-Lig-1@40 °C (Figure 7c), approximately 90% inhibition was obtained at 4 mg/mL (∼4 log inhibition). The intact sponges displayed insignificant bacterial adsorption; however, when the sponges were seized up into smaller pieces (Figure 7d), complete inhibition was obtained at 4 mg/mL, due to their highly porous structure as revealed previously in the SEM images. There were no viable bacteria detected from the supernatant, confirming the complete bacterial adsorption. This is due to an increase in the surface area and therefore increased contact-dependent adsorption of *E. coli*. From this experiment, the dependence of antibacterial properties on the dosage was confirmed.

## 4 Conclusion

The lignin–chitosan hybrid materials synthesized in this work present a multifunctional system capable of removing both synthetic dyes and microbial contaminants from aqueous media. The synergistic combination of the aromatic structure of lignin and chitosan’s cationic nature enables efficient adsorption of dye molecules through electrostatic interactions and π–π stacking. This also allows the simultaneous capture and potentially inactivation of bacteria via surface charge interactions. The maximum adsorption capacities of the lignin–chitosan composites reached 44 wt % and follow the Langmuir model of adsorption. When the material was fragmented into smaller particles, a pronounced antibacterial effect was observed at lower concentrations compared with the intact bulk form. This suggests that reducing the particle size enhances the available surface area and active site accessibility, thereby improving antibacterial performance. Furthermore, the mechanical robustness of spherical beads makes them suitable for use in fixed-bed column configurations, offering a scalable and sustainable approach to continuous-flow wastewater treatment. This work opens future avenues for evaluating breakthrough behavior, antimicrobial retention efficiency, and reuse potential under dynamic flow conditions.

## Supplementary Material



## Notes

Characterization of chitosan, characterization of kraft lignin, characterization of lignin–chitosan composites, EDC/NHS coupling of chitosan with carboxylated lignin, dye adsorption, adsorption isotherms, adsorption kinetics, and adsorption of methylene blue (PDF)
