# Supplementary material for: Upcycling Lignin into Porous Hybrid Beads and Sponges for Efficient Removal of Organic and Biological Contaminants from Water
Source: ACS Mater Au. 2025 Dec 9;6(2):347–63. doi: 10.1021/acsmaterialsau.5c00147 (PMC12983099; doi:10.1021/acsmaterialsau.5c00147)
Supplement: Supplementary file 1 [file mg5c00147_si_001.pdf]

Supporting Information

Upcycling Lignin into Porous Hybrid Beads and Sponges for Efficient Removal of Organic and Biological Contaminants from Water

Nicholas Breitkreuz[a], Tatyana L. Povolotsky[a], Philip Nickl[a], Mohsen Adeli[a,b], Rainer Haag[a]*, Sanjam Chandna[a]*

a Institute for Chemistry and Biochemistry, Freie Universität Berlin, 14195 Berlin, Germany

b Department of Organic Chemistry, Faculty of Chemistry, Lorestan University, 68151-44316, Iran

Corresponding author, Email: haag@zedat.fu-berlin.de, sanjam1994@zedat.fu-berlin.de

Table of Contents

S1. Characterisation of Chitosan 2

S2. Characterisation of Kraft Lignin 4

S3. Characterisation of lignin-chitosan composites 7

S4. EDC/NHS Coupling of Chitosan with Carboxylated Lignin 8

S5. Dye adsorption 10

S6. Adsorption Isotherms 11

S7. Adsorption Kinetics 13

S8. Adsorption of Methylene Blue 14

S1. Characterisation of Chitosan

The modification of chitosan, specifically the amine moieties present within chitosan particles, is of interest with regarding to the formation of lignin-chitosan composites. The deacetylation degree (DD) describes the amount of D-glucosamine present within chitosan; therefore, to obtain the desired lignin-chitosan products, it is crucial to first determine the DD to get the optimal stoichiometric ratio between the biopolymers for the reactions.

A calibrated electrode connected to a pH meter was used to track the change in pH over the course of the titration. The chitosan was first dissolved in HCl to a pH of approx. 1.8. It was then titrated against 0.1 M NaOH (Figure S1 – Supplementary Information) until the pH of the solution reached a value of 12. Throughout the course of the titration, it was possible to identify two separate equivalence points; the first equivalence point at pH 3.89 corresponds to the neutralisation of the excess HCl (Eq 1), whereas the second equivalence point at pH 8.8 corresponds to the deprotonation of GlcN subunits (Eq 2).

$$H^+ + OH^- \rightarrow H_2O \qquad (Eq\ 1)$$

$$R - NH_3^+ + OH^- \rightarrow R - NH_2 + H_2O \qquad (Eq\ 2)$$

From Figure S1a it could be observed that the second equivalent point is not well defined as the first derivative does not yield a narrow peak. This is caused by the precipitation of chitosan beyond pH>6. Not only does the precipitation of chitosan results in lower chitosan concentration in solution, but the precipitated chitosan also covered the surface of the electrode, thereby inducing errors in the value of the measured pH. To mitigate the effects of these errors on the final calculation, only the results until the solution reached a pH value of 3 were used to determine the linear relationship between the function f(x) and the volume of NaOH added (Figure S1b – Supplementary Information). The volume of NaOH required to reach the endpoint of the titration could then be determined by extrapolation of the linear titration curve to the x-axis. Through Eq. 5-7, the DD of the chitosan was determined to be 88%.

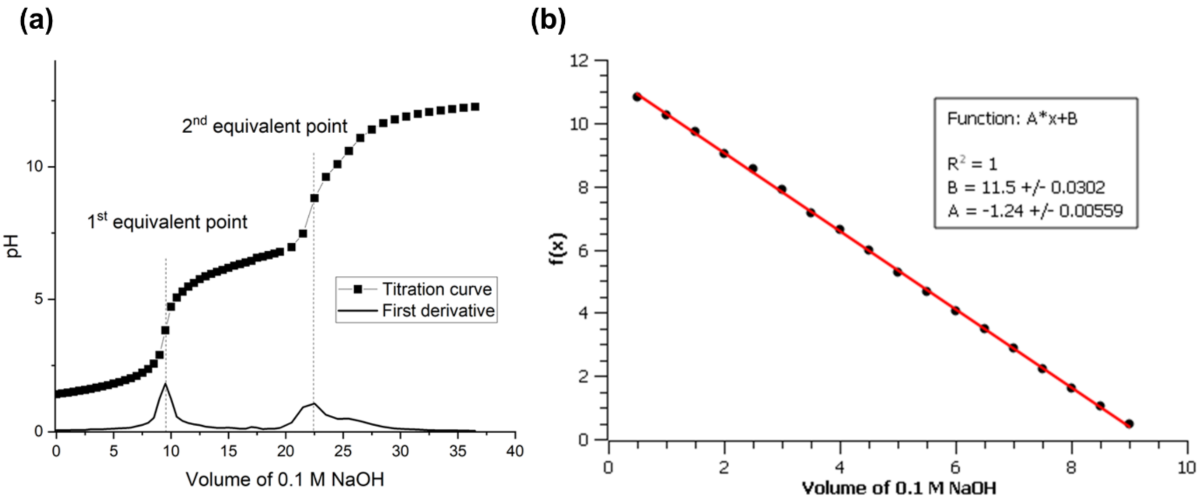

Figure S1. (a) Titration curve of chitosan. Two observable equivalence points could be seen from its first derivative. (b) Linear curve from plotting the function f(x) vs volume of NaOH.

Table S1. DLS measurements for chitosan and kraft lignin.

	Hydrodynamic Radius [nm]	Đ
Chitosan	868.5	0.744
Lignin	421.5	0.336

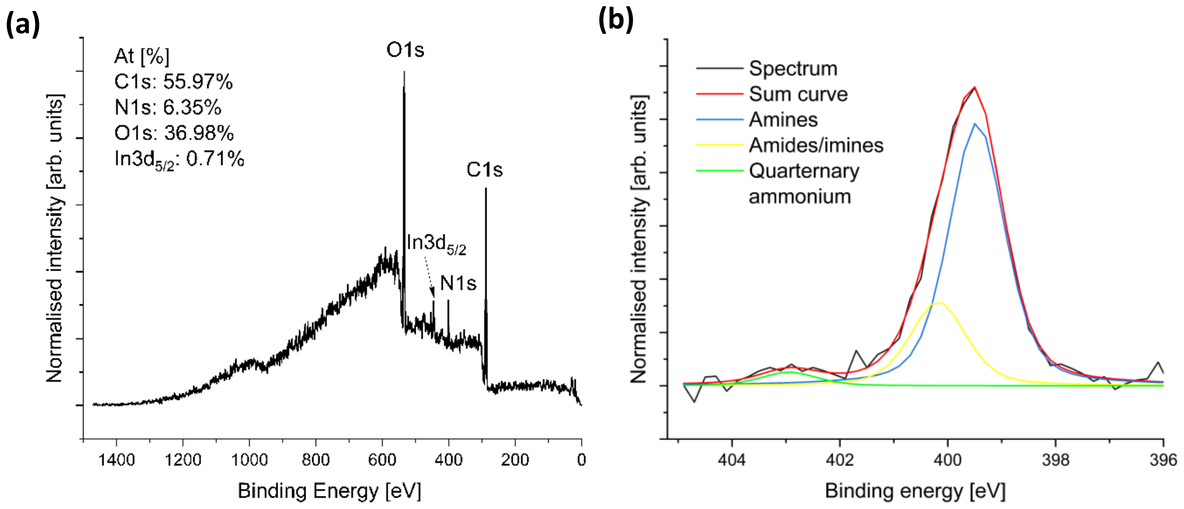

Figure S2. XPS spectra of chitosan measured on an indium foil. (a) Survey spectrum (indium foil signals were detected due to X-ray surface penetration) (b) highly- resolved N1s elemental spectrum.

Table S2. Summary of results from N1s spectra of chitosan.

	Binding energy [eV]	Rel. area
Amines	399.4611	0.7289
Amides/imines	400.1814	0.2337
Quarternary ammonium	402.9416	0.0374

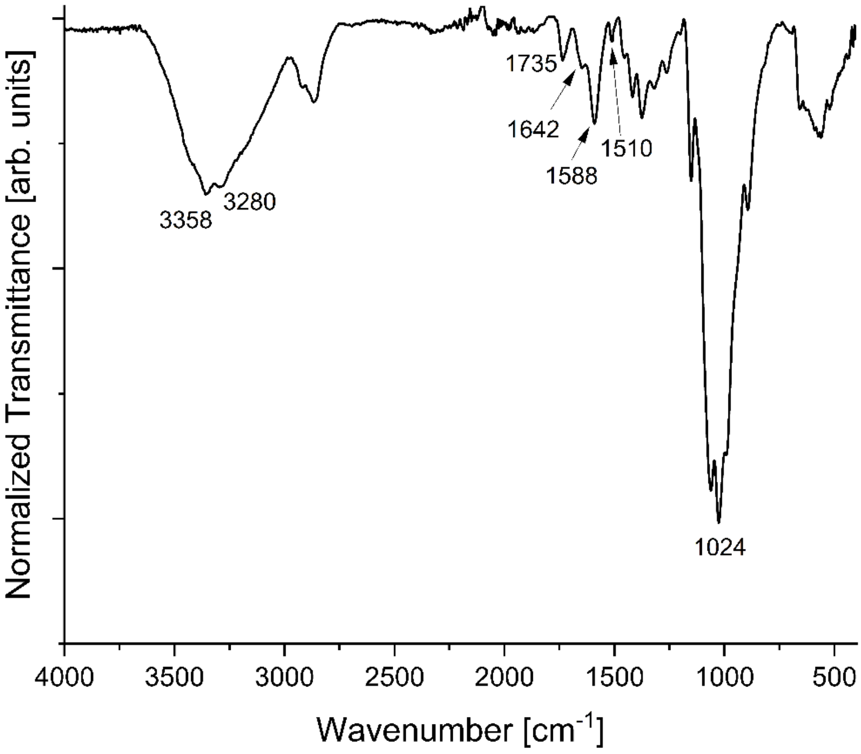

Figure S3. FTIR spectrum of chitosan

S2. Characterisation of Kraft Lignin

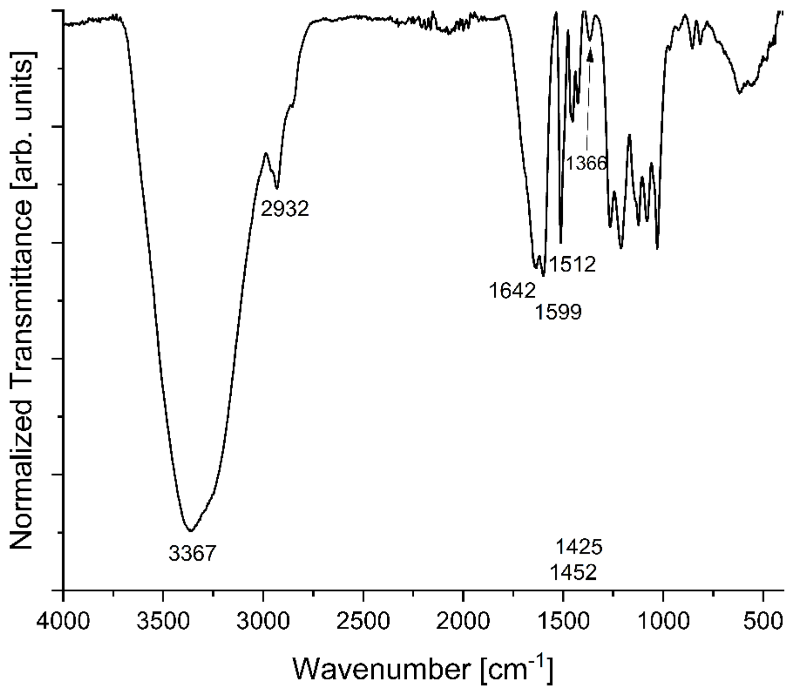

Figure S4. FTIR spectrum of KL

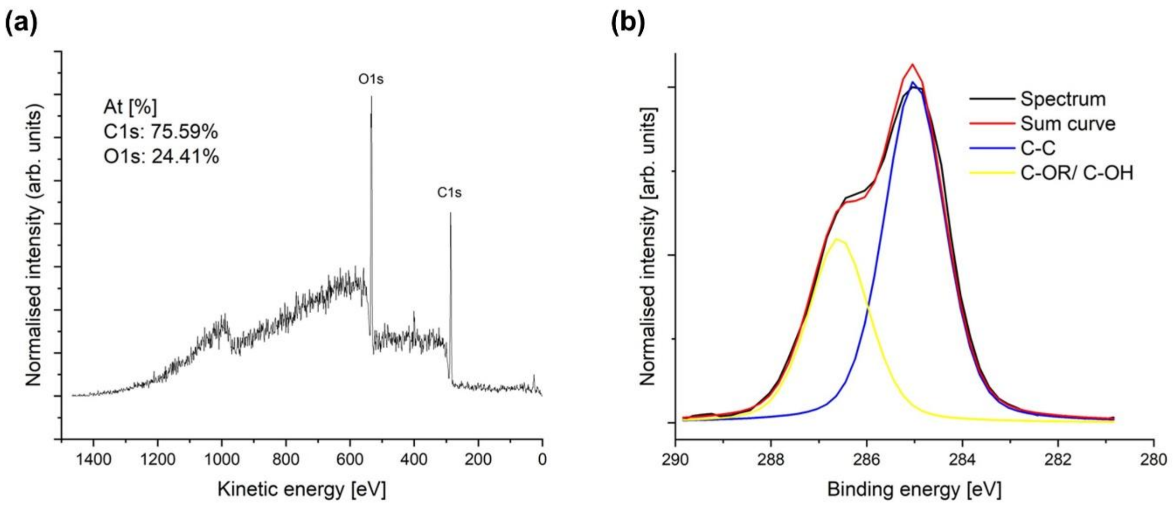

Figure S5. XPS spectra of Kraft lignin. (a) Survey spectrum, (b) highly- resolved C1s elemental spectrum.

Table S3. Summary of results from C1s spectra of kraft lignin.

	Binding energy [eV]	Rel. area
C-C	285.0000	0.6496
C-OR/ C-OH	286. 5773	0.3504

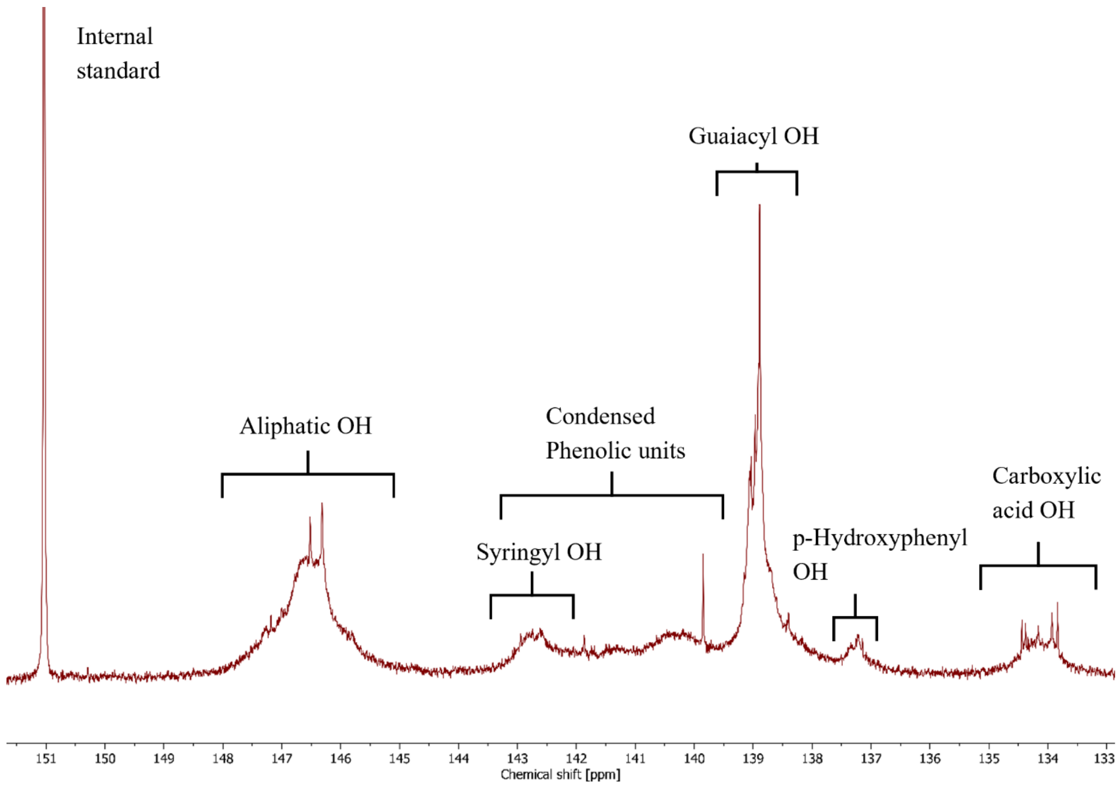

Figure S6. ^{31}P NMR of kraft lignin.

Table S4. Quantification of signals from the ^{31}P NMR of the kraft lignin

Signal	Chemical shift [ppm]	Integral value	Quantity [mmol/g]
Internal standard	151.8	1.00	N/A
Aliphatic OH	150.0-144.6	1.87	2.32
Phenolic OH	144.6-137.0	3.19	3.95
Condensed phenolic OH (C5-substituted)	144.6-140.4	1.38	1.71
β-5 phenolic units	144.6-143.0	0.44	0.54
Syringyl phenolic OH	143.0-141.8	0.36	0.44
5-5′ biphenyl phenolic units	141.8-140.4	0.58	0.72
Guaiacyl phenolic OH	140.4-138.3	1.61	2.00
p-Hydroxyphenyl phenolic OH	138.3-137.0	0.20	0.25
Carboxylic acid	136.0-133.6	0.43	0.53
Total OH	151.2-133.2	5.59	6.92

S3. Characterisation of lignin-chitosan composites

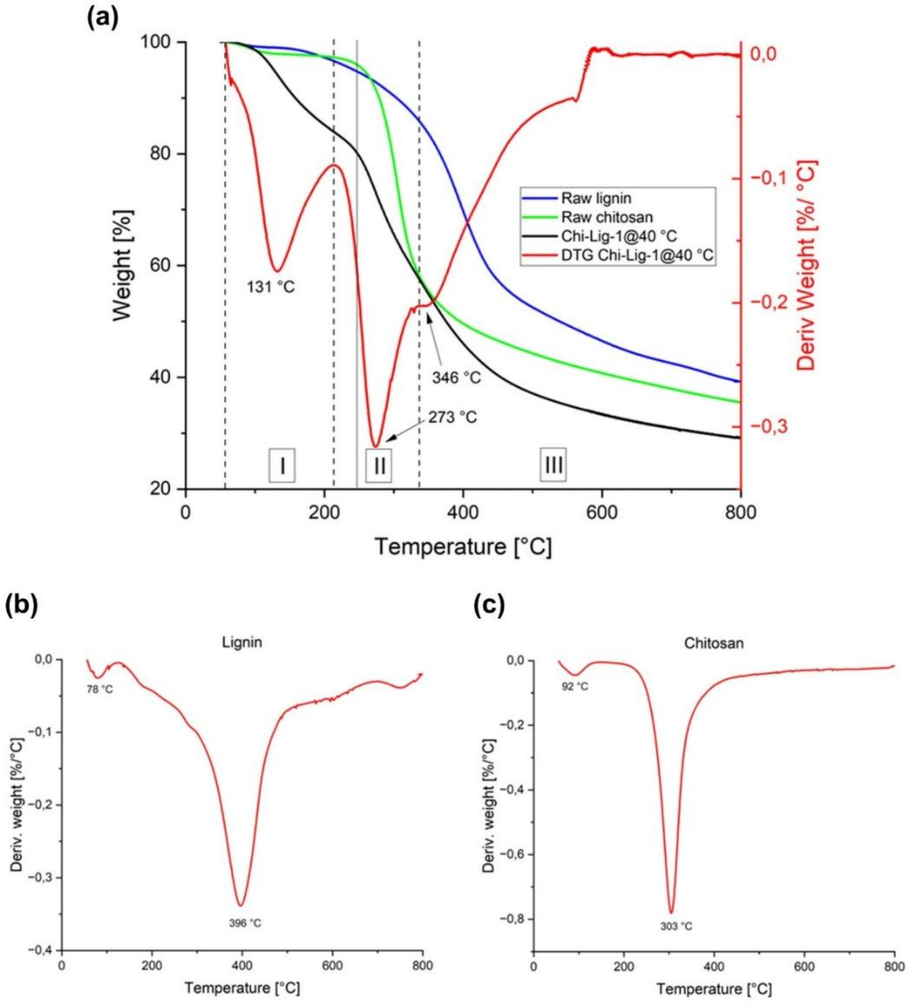

Figure S7. TGA of (a) Chi-Lig-1@40 °C, (b) chitosan, and (c) lignin.

(a)

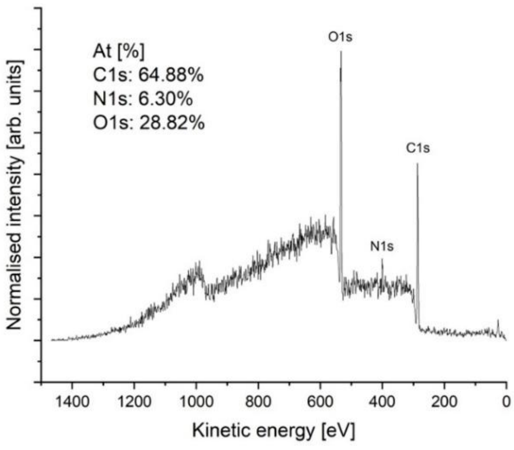

(b)

	Binding energy [eV]	Rel. area
**C1s**		
C-C	285.035	0.4074
C-OR/ C-OH	286.6209	0.4748
C=O	288.1424	0.1178
**N1s**		
Amines	399.2055	0.4787
Amides/ imines	400.2046	0.2826
Charge	401.7658	0.2387

Figure S8. XPS results for Chi-Lig-1@40 °C. a) Survey spectrum. b) Summary of results from N1s and C1s spectra.

S4. EDC/NHS Coupling of Chitosan with Carboxylated Lignin

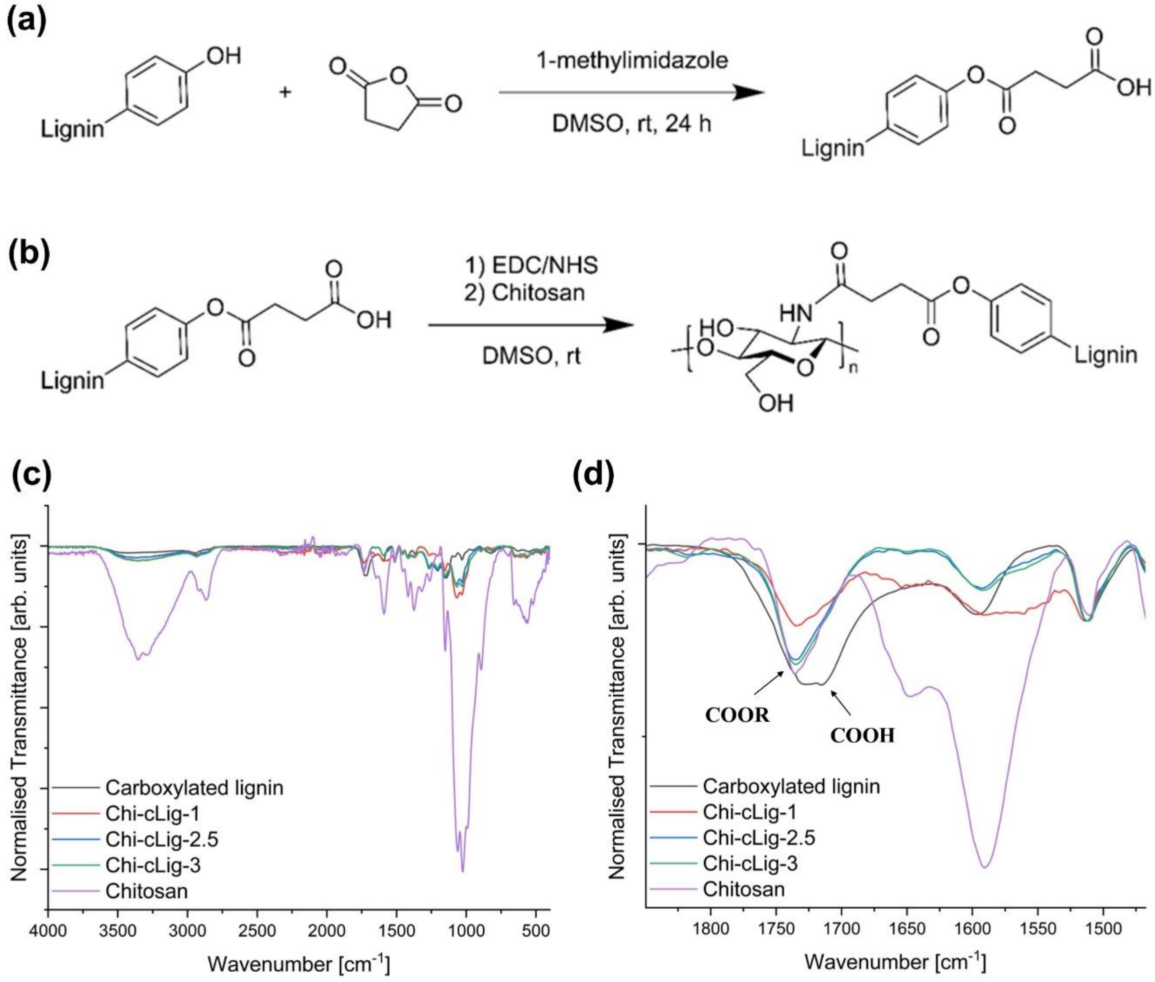

Figure S9. Chemical scheme of a) functionalisation of lignin using succinic anhydride and b) EDC/NHS coupling of carboxylated lignin with chitosan. FTIR spectrum of c) carboxylated lignin, Chi-cLig composites and chitosan and d) carboxylated lignin, Chi-cLig composites and chitosan (zoomed in at 1475 cm^{-1} to 1850 cm^{-1}).

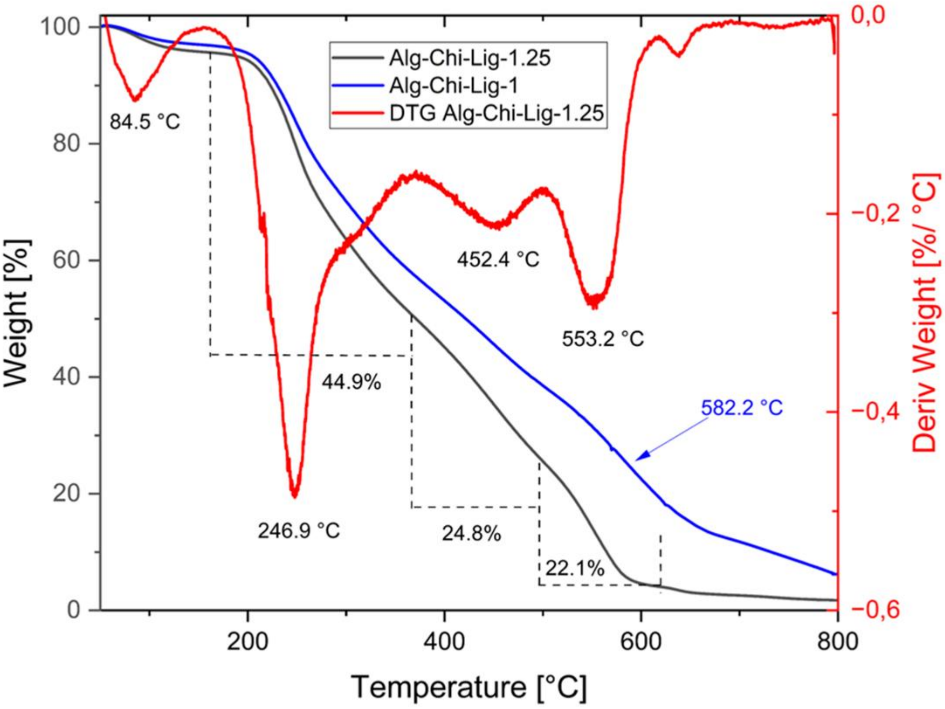

Figure S10. TGA of Alg-Chi-Lig-1 and Alg-Chi-Lig-1.25 beads.

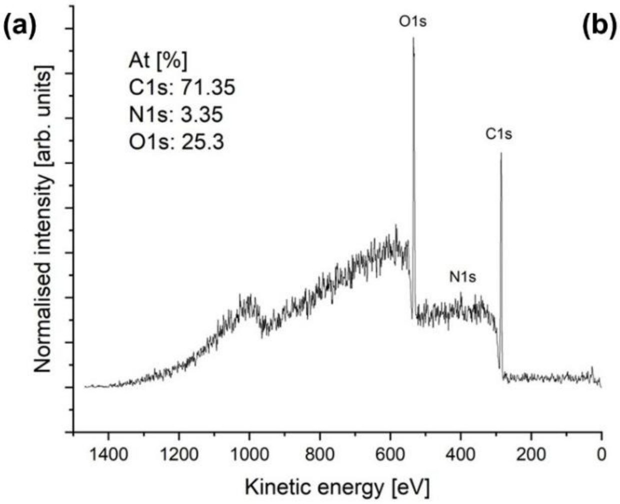

(b)

	Binding energy [eV]	Rel. area
**C1s**		
C-C	285.0427	0.498
C-OR/ C-OH	286.6209	0.3801
C=O	287.9775	0.1219
**N1s**		
Amines	399.0811	0.4362
Amides/ imines	399.9007	0.3244
Charge	402.2939	0.2394

Figure S11. XPS results for G-Chi-Lig-66%. a) Survey spectrum. b) Summary of results from N1s and C1s spectra.

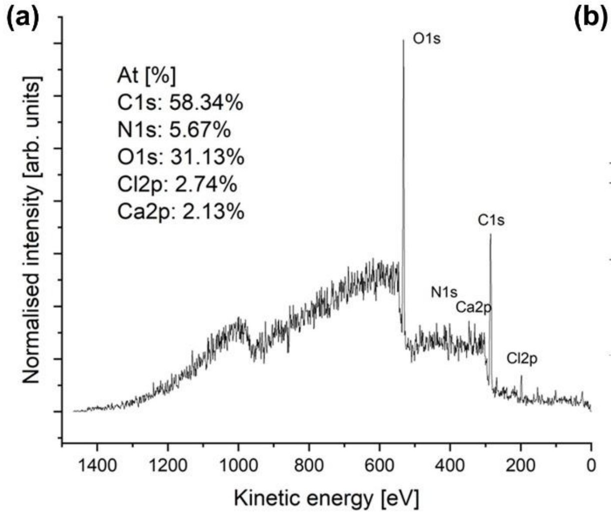

(b)

	Binding energy [eV]	Rel. area
**C1s**		
C-C	284.9725	0.3316
C-OR/ C-OH	286.5026	0.4656
C=O	288.1626	0.143
**N1s**		
Amines	399.6506	0.2915
Amides/ imines	401.1369	0.3589
Charge	402.0024	0.3497

Figure S12. XPS results for Alg-Chi-Lig-1. a) Survey spectrum. b) Summary of results from N1s and C1s spectra.

S5. Dye adsorption

The morphology of Alg-Chi-Lig-1 before and after adsorption with methyl orange underwent massive changes. Prior to dye adsorption, the pristine bead displayed a relatively smooth surface with no observable pores. After adsorption with methyl orange, the surface became rough and wrinkled due to deposition of dye molecules on the surface of the bead. Furthermore, a reduction in size of the bead was observed. A potential explanation for the shrinkage could be that the interaction of the dye with the alginate causes further crosslinking within the structure, thus it became more compact.

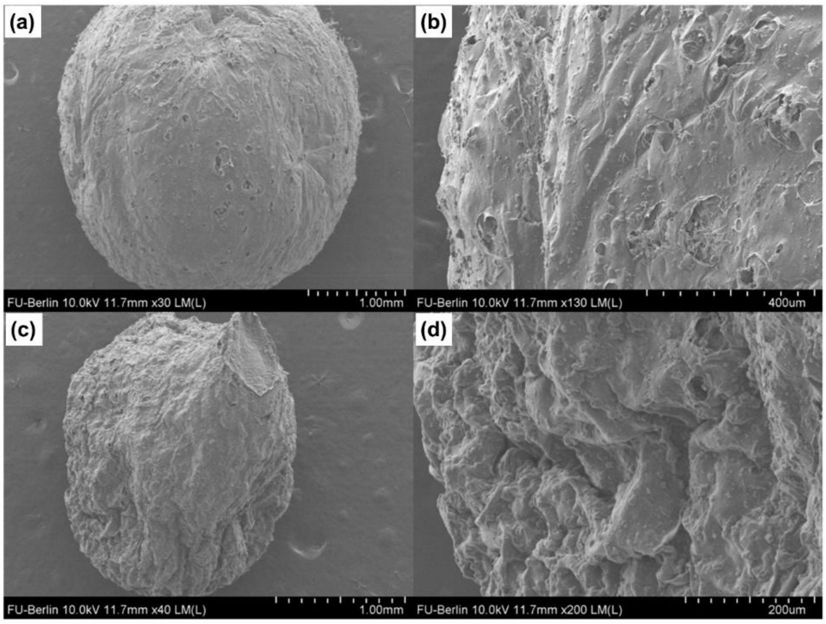

Figure S13. SEM images of Alg-Chi-Lig-1 beads at different magnifications. (a-b) before methyl orange adsorption and (c-d) after methyl orange adsorption.

S6. Adsorption Isotherms

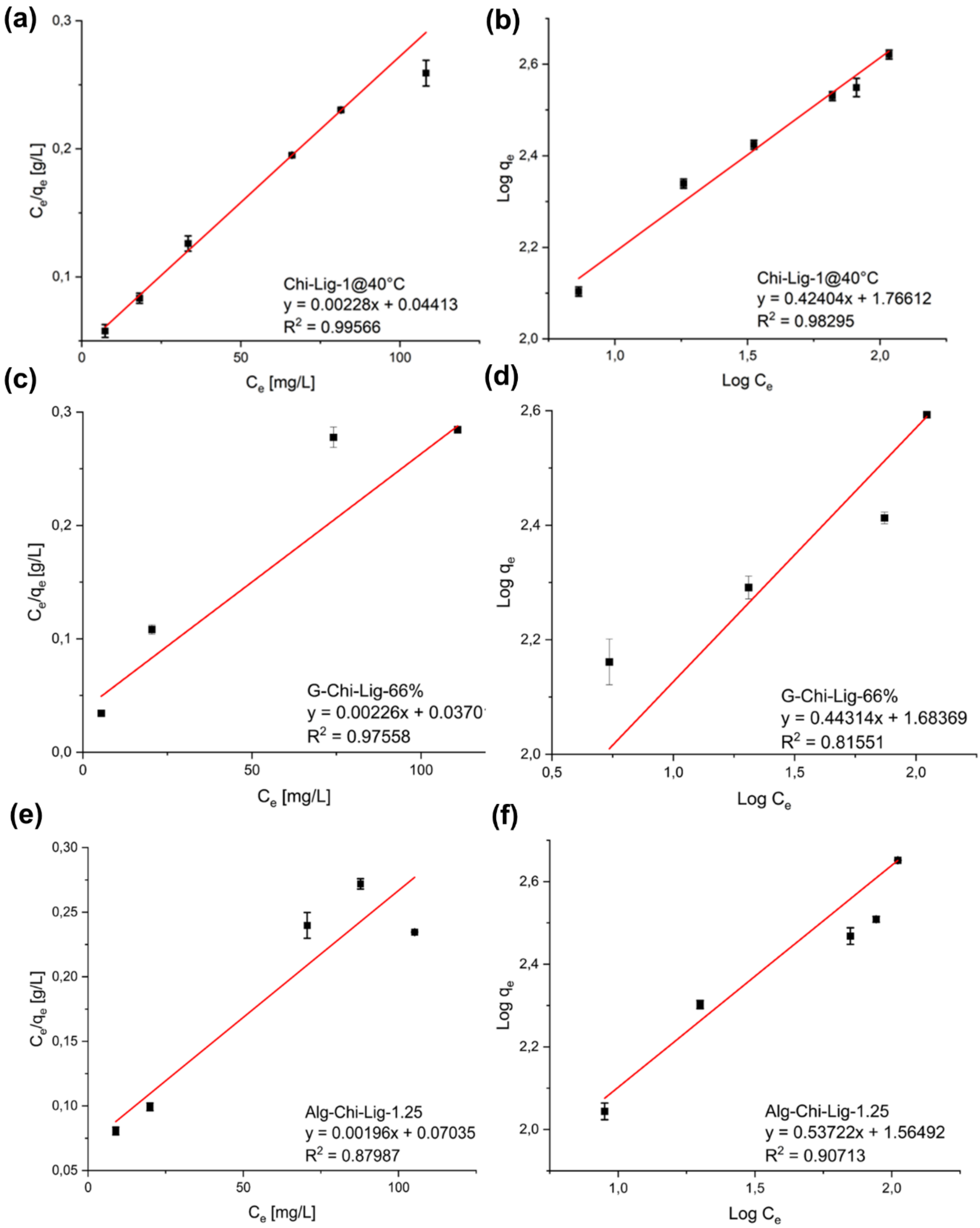

Figure S14. Adsorption isotherms for the adsorption of lignin-chitosan composites for methyl orange. (Left) Linearised Langmuir isotherm. (Right) Linearised Freundlich isotherm.

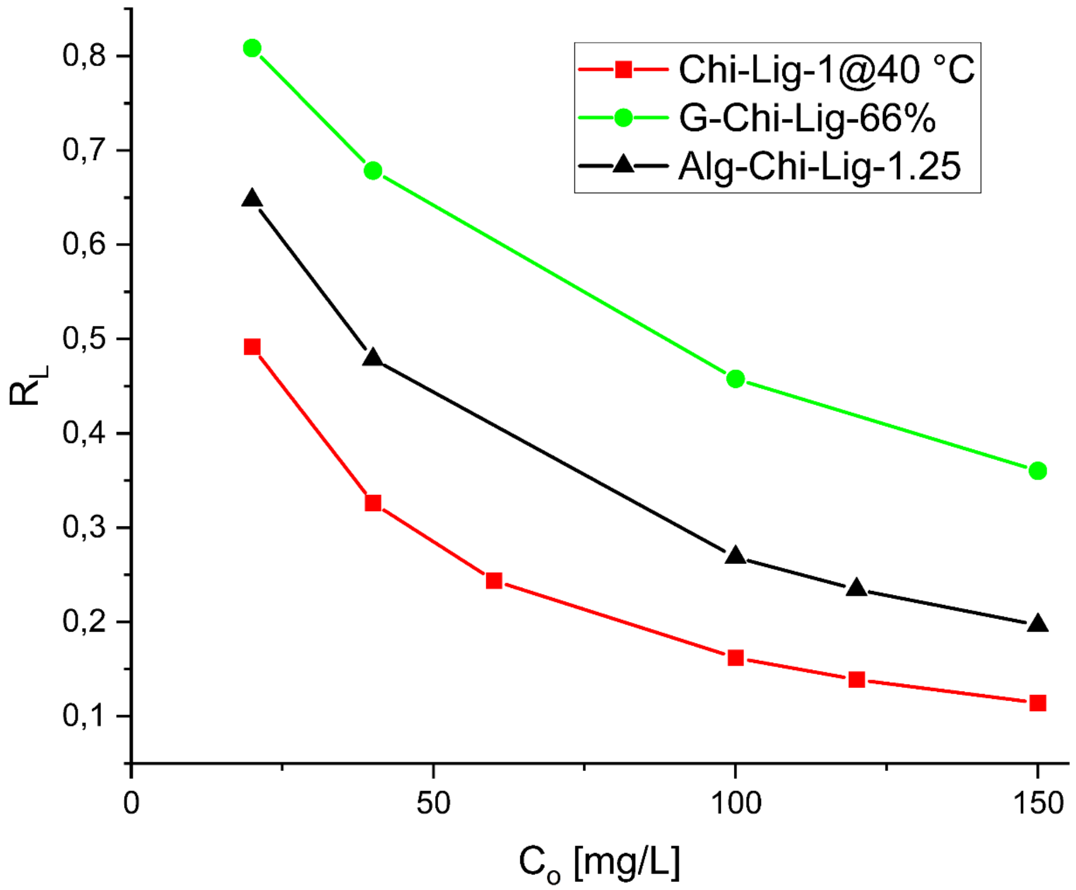

Figure S15. RL values of lignin-chitosan composites at different initial methyl orange concentrations.

S7. Adsorption Kinetics

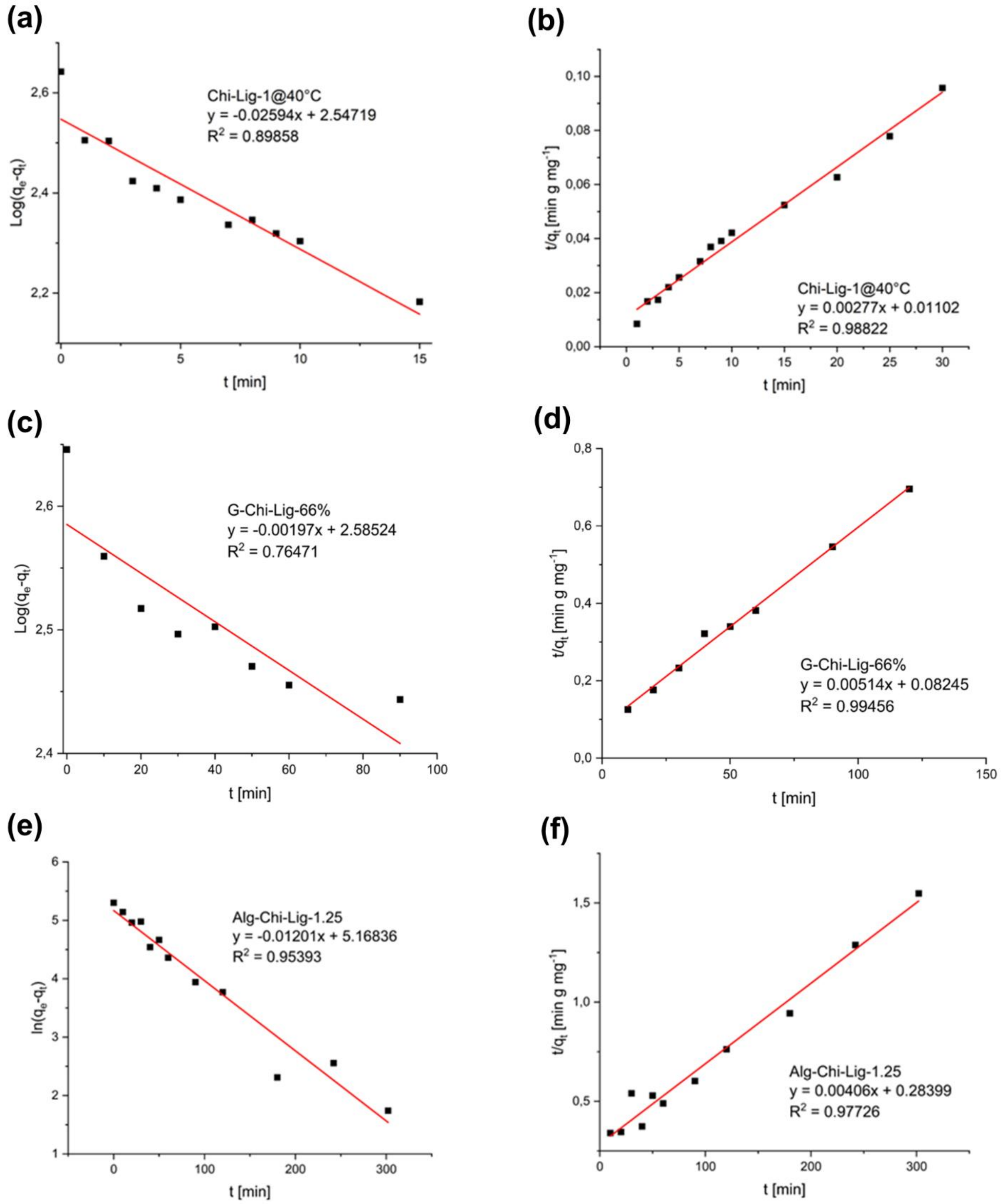

Figure S16. (Left) Pseudo-first order kinetics (PFO) (Right) Pseudo-second order kinetics (PSO) of methyl orange adsorption.

Table S5. kinetic rate constants and maximum adsorption capacity of methyl orange by lignin-chitosan composites, as determined by PFO and PSO models.

	PFO				PSO		
Sample	[a]q_{eexp} [mg/g]	[b]q_{ecalc} [mg/g]	k_1 [min^{-1}]	R^2	q_{eexp} [mg/g]	k_2 [min^{-1}]	R^2
Chi-Lig-1@40 °C	352.5	438.6	0.060	0.89858	361.0	$6x10^{-4}$	0.98822
G-Chi-Lig-66%	384.8	442.5	0.005	0.76471	194.6	$3x10^{-4}$	0.99456
Alg-Chi-Lig-1.25	454.7	510.2	0.002	0.80686	246.3	$6x10^{-5}$	0.97726

S8. Adsorption of Methylene Blue

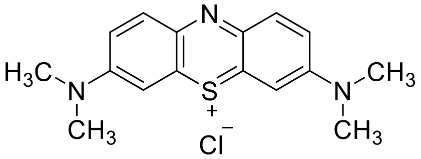

Scheme 1. Chemical structure of methylene blue (MB).

The adsorption of methyl orange to lignin-chitosan composites could be enabled through either hydrogen bonding, π- π interactions or electrostatic interactions. To determine if electrostatic interaction was the principal interaction that governed the adsorption behaviour of the composites, the adsorption of methylene blue (MB) was tested. Unlike methyl orange, methylene blue is an cationic dye where the positive charge is delocalised throughout the molecule. It was chosen due to its similar molecular weight and structure to methyl orange. Figure S16 shows the decolorization efficiency of all three types of lignin-chitosan composites for methylene blue. Maximum adsorption was achieved using raw lignin as well as alginate containing beads, whereas other materials barely removed any dye from solution. For all composite materials, the low affinity for methylene blue compared to methyl orange suggests that electrostatic interactions dominate the adsorption process, as the major difference separating the 2 dyes is its charges. The highest decolourisation efficiency was observed for lignin as it has the most negative surface charges. Slightly worse performance was shown by Alg-Chi-Lig-x beads, which removed only 6-7% of dyes from the solution. The incorporation of sodium alginate was known to facilitate greater adsorption affinity due to increased hydroxyl and carboxyl contents. Lastly, G-Chi-Lig-66% and Chi-Lig-1 essentially did not remove any MB from solution (<2% decolorisation efficiency).

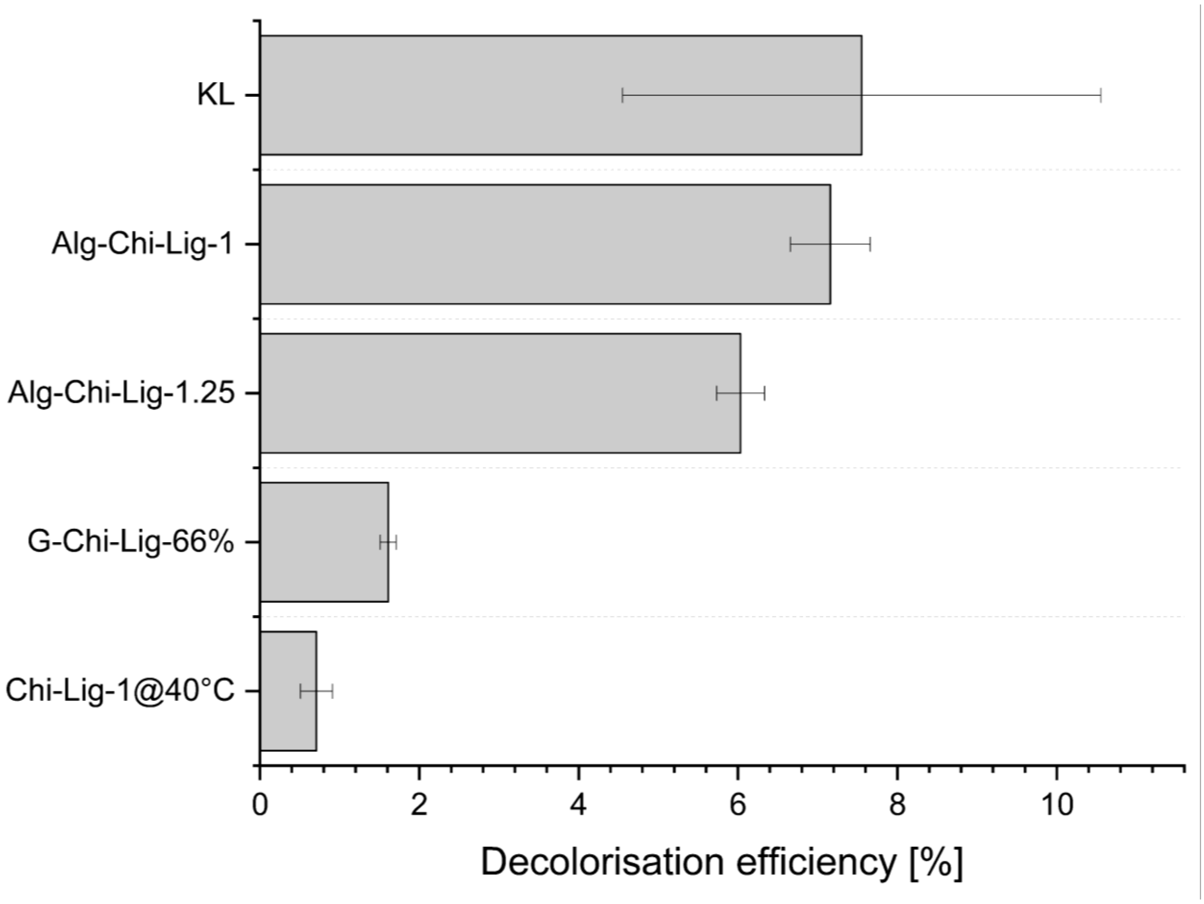

Figure S17. Decolorisation efficiency of lignin-chitosan composites for the removal of MB from aqueous solution.
